# Genome-wide association studies of ionomic and agronomic traits in USDA mini core collection of rice and comparative analyses of different mapping methods

**DOI:** 10.1186/s12870-020-02603-0

**Published:** 2020-09-24

**Authors:** Shuai Liu, Hua Zhong, Xiaoxi Meng, Tong Sun, Yangsheng Li, Shannon R. M. Pinson, Sam K. C. Chang, Zhaohua Peng

**Affiliations:** 1 Department of Biochemistry, Molecular Biology, Entomology and Plant Pathology, Mississippi State University, Starkville, MS 39762 USA; 2 State Key Laboratory of Hybrid Rice, Key Laboratory for Research and Utilization of Heterosis in Indica Rice, Ministry of Agriculture, College of Life Sciences, Wuhan University, Wuhan, China; 3 Dale Bumpers National Rice Research Center, USDA ARS, Stuttgart, AR 72160 USA; 4 Experimental Seafood Processing Laboratory, Coastal and Research Extension Center, Mississippi State University, Pascagoula, MS 39567 USA

**Keywords:** Rice, Ionomic traits, Agronomic traits, Multivariate GWAS

## Abstract

**Background:**

Rice is an important human staple food vulnerable to heavy metal contamination leading to serious concerns. High yield with low heavy metal contamination is a common but highly challenging goal for rice breeders worldwide due to lack of genetic knowledge and markers.

**Results:**

To identify candidate QTLs and develop molecular markers for rice yield and heavy metal content, a total of 191 accessions from the USDA Rice mini-core collection with over 3.2 million SNPs were employed to investigate the QTLs. Sixteen ionomic and thirteen agronomic traits were analyzed utilizing two univariate (GLM and MLM) and two multivariate (MLMM and FarmCPU) GWAS methods. 106, 47, and 97 QTLs were identified for ionomics flooded, ionomics unflooded, and agronomic traits, respectively, with the criterium of *p*-value < 1.53 × 10^− 8^, which was determined by the Bonferroni correction for *p*-value of 0.05. While 49 (~ 20%) of the 250 QTLs were coinciding with previously reported QTLs/genes, about 201 (~ 80%) were new. In addition, several new candidate genes involved in ionomic and agronomic traits control were identified by analyzing the DNA sequence, gene expression, and the homologs of the QTL regions. Our results further showed that each of the four GWAS methods can identify unique as well as common QTLs, suggesting that using multiple GWAS methods can complement each other in QTL identification, especially by combining univariate and multivariate methods.

**Conclusions:**

While 49 previously reported QTLs/genes were rediscovered, over 200 new QTLs for ionomic and agronomic traits were found in the rice genome. Moreover, multiple new candidate genes for agronomic and ionomic traits were identified. This research provides novel insights into the genetic basis of both ionomic and agronomic variations in rice, establishing the foundation for marker development in breeding and further investigation on reducing heavy-metal contamination and improving crop yields. Finally, the comparative analysis of the GWAS methods showed that each method has unique features and different methods can complement each other.

## Background

Rice is an important cereal which feeds more than half the world’s population [1]. With the rapid expansion of global population, food security has become a highly challenging task. Meanwhile, anthropogenic activities such as mining, smelting, chemical engineering, energy related industry, and broad application of pesticides & fertilizers in agriculture have led to frequent heavy metal contamination in soil, including Cadmium (Cd), Manganese (Mn), Nickel (Ni), and metalloid Arsenic (As) [2]. Soil with excessive heavy metal elements represses plant germination and growth, resulting in a decrease of crop yield [3, 4]. Meanwhile, plants uptake the toxic heavy metal elements from contaminated soil and accumulate them in edible plant tissues, leading to food contamination.

The anaerobic growing conditions of flooded rice paddies and the unique physiology of the rice plant allow rice to take up some heavy metals from water and soils in a highly efficient manner and sequester it in different organs within the plants, including the grains consumed by humans. The arsenic concentration in rice grains is roughly about 10 times higher than other crops grown in the same region even if the soil has no or limited anthropogenic contamination [5]. Rice has been reported to contribute substantially to inorganic and organic arsenic [6–8] intake by the human population in many regions of the world. As was ranked on the top of the US Agency for Toxic Substances and Disease Registry (ATSDR) Priority List of Hazardous Substances since 1997 (https://www.atsdr.cdc.gov/spl/index.html#2017spl). It has also been listed as a toxic component by many other countries and treated as a critical contaminant during food safety inspection. Cd is one of the most toxic heavy metals, and can easily reach the food chain due to strong assimilation by crops [9, 10]. Once absorbed, Cd is efficiently retained in the human body and may cause it to stay throughout the life span with an estimated half biology life between 6 to 38 years in kidney and between 4 to 19 years in the liver (ATSDR, 1999).

In contrast to heavy metals, many mineral elements are essential to humans but deficient in rice grains, for example, zinc, calcium, and iron [11]. Increasing the concentrations of these minerals can improve the nutritional value of rice thus promoting human health for those consuming rice as the staple food. However, it is highly challenging to either increase the essential minerals or reducing the heavy metal due to lack of understanding of the genetic bases and molecular mechanisms of the related traits. Further, it is still poorly explored whether the concentration of mineral or heavy metal is associated with agronomic traits. Although there are multiple rice association mapping studies with specific minerals, heavy metals, and agronomic traits, respectively, these studies used either different mapping populations or different statistical analyses [12–14]. Therefore, each of the studies revealed some but not all facets of the genetic bases of rice variations. Recent accessibility to comprehensive sequence data, and the development of software facilitating the use of more powerful statistical analytics, opens the opportunity for more comprehensive study and understanding of the genetic bases of these traits.

The USDA Rice Core Collection, containing about 10% of the whole NSGC (National Small Grains Collection) Rice Collection, was assembled by stratified random sampling method in 2002, which has been evaluated comprehensively for 25 characteristics and proven to be highly representative of the whole collection [15]. The Rice Mini-Core Collection contains approximately 10% of the Core Collection [16]. The grain mineral concentrations have been analyzed under flooded and unflooded growth conditions [17], and the agronomic traits have also been evaluated for the Core Collection [18]. But most of these researches were done before the genome sequencing data was available.

Biparental genetic mapping and Genome-wide-association-study (GWAS) are the two different tools for mapping Quantitative Trait Locus (QTL). GWAS involves studying natural populations with greater historical recombination events, which could detect more QTLs from broader genetic variation [19]. Therefore, it can explore genetic resources that cannot be revealed by studying the offspring of biparental crosses in linkage mapping [20]. GWAS has been applied successfully to a variety of plants, including *Arabidopsis* [21], maize [22, 23], barley [24], wheat [25], rice [26, 27], soybean [28], and cotton [29]. It is a critical tool for crop improvement.

Univariate GWAS is a mapping method that has been successfully used for gene mapping in plants and animals. However, a large number of genes may not be detected (false negative QTLs) due to the confounding problems between population structure, kinship, and markers. The population structure causes genome-wide linkage disequilibrium between unlinked loci, which leads to statistical confounding in genome-wide association studies. Mixed models have been shown to deal well with the confounding effects of a large number of small effect loci in the diffusion background, but they do not always account for large effect loci [30]. Multivariate GWAS method considers the confounding problem between covariates and test marker to detect more QTLs and previous reports showed that multivariate GWAS had lower FDR when using the same threshold compared with univariate GWAS method [30]. In recent years, a large number of multivariate GWAS methods have been developed, including MLMM (multi-locus mixed-model) [30], FarmCPU (Fixed and random model Circulating Probability Unification) [31], mrMLM (multi-locus random-SNP-effect MLM) [32], FASTmrMLM (fast mrMLM) [33], FASTmrEMMA (fast multi-locus random-SNP-effect efficient mixed model analysis) [34], pLARmEB (polygenic-background-control-based least angle regression plus empirical Bayes) [35], pKWmEB (integration of Kruskal-Wallis test with empirical Bayes) [36], ISIS EM-BLASSO (iterative modified-sure independence screening expectation-maximization-Bayesian least absolute shrinkage and selection operator) [37], and GPWAS (Genome-Phenome Wide Association Study) [38]. The MLMM [30] uses forward-backward stepwise linear mixed-model regression, forward stepwise uses the most significant associated SNP as a new fixed-effect covariate (cofactor) and creates a new model until reaching a pre-specified maximum number of forward steps, backward stepwise means to remove least significant SNP and create a new smaller model until only one selected marker is left. Whereas, FarmCPU [31] performs marker tests with associated markers as covariates in a Fixed Effect Model (FEM), and then optimization on the associated covariate markers in a Random Effect Model separately. These multivariate GWAS methods were successfully applied to several different crop species, including cotton [39], rice [40, 41], foxtail millet [42], soybean [43, 44], maize [45, 46], and wheat [47, 48].

GWAS has been widely used for rice mapping research. However, most of the studies used univariate methods. The multivariate analysis methods and the deep DNA sequencing resources are still poorly explored. For example, Yan et al.(2014) [49] performed limited GWAS on agronomic traits using only 155 molecular markers (154 SSR makers and one indel marker). However, the related loci could not be precisely defined in the study due to limited number of markers. With the development of the sequencing technology, more markers, especially SNP markers, become available and have been shown to be successful in association mapping studies [50, 51]. The resequencing data of the mini core has been deposited to SRA database https://www.ncbi.nlm.nih.gov/sra (Accession: PRJNA301661) recently [52], which opened an opportunity for us to improve GWAS with higher density of genotypic data and evaluate the validity of different GWAS analysis methods. In order to identify all possible QTLs controlling the ionomic and agronomic traits for marker development and gene characterization, we employed two univariate GWAS methods (GLM and MLM) and two multivariate GWAS methods (MLMM and FarmCPU) to detect the related QTLs in USDA rice mini core collection. The analyzed ionomics traits included As, Ca, Co, Cd, Cu, Fe, K, Mg, Mn, Mo, Ni, P, Rb, S, Sr, and Zn) and the agronomic traits involved amylose [AMYLOSE], awn type [AWNTYPE], flowering time [DAYSFLOWER], hull color [HULLCOLOR], hull cover [HULLCOVER], kernel length [KERNELLEN], kernel width [KERNELWID], kernel rate [KERNELRAT], kernel weight [KERNELWT], lodging [LODGING], panicle type [PANICLETYPE], plant height [PLANTHT], and plant type [PLANTTYPE]). Our results showed that we successfully remapped 49 loci/genes which have been shown to play essential roles in rice agronomic and ionomic trait control. Meanwhile, over 200 novel loci involved in heavy metal, minerals, and agronomic traits control were discovered. In addition, multiple candidate genes involved in ionomic and agronomic traits control were identified via DNA sequence, expression, and homologous analyses. These studies provided novel insights into the genetic basis of ionomic and agronomic trait variations in rice and possible correlations among these traits. The results will have critical reference value in further fine mapping the related genetic loci and in guiding rice breeding.

## Results

### Characteristics of SNPs

**Fig. 1:**
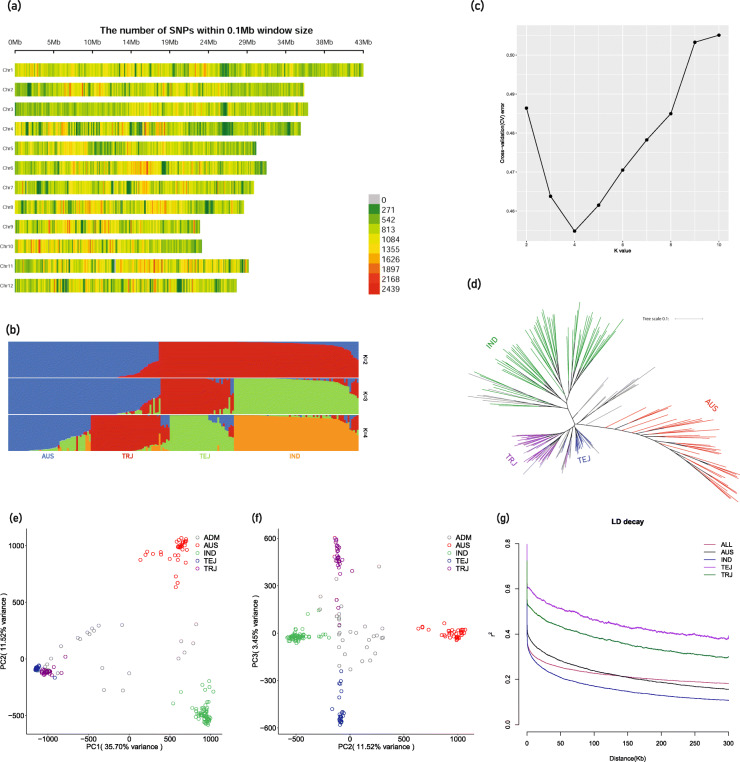
Sequence and structure analysis of USDA mini core collection. **a** Distribution of SNPs on the rice chromosomes. Number of SNPs per 0.1 Mb window was shown as a color index (bottom right), **b** Ancestries analysis for each individual was inferred using admixture, **c** Cross-validation error (CV) score across different K value. The best K value (K = 4) was chosen according to the lowest CV score for the admixture analysis, **d** Phylogenetic tree of 191 rice accessions. Green indicated Indica (IND) rice, Red indicated Aus (AUS) rice, Purple represented Tropical Japonica (TRJ) rice; Blue represented Temperate Japonica (TEJ) rice, **e** PCA showing genetic variation in the rice accessions with first and second PCs, the color was defined by current Admixture analysis. **f** PCA showing genetic variation in the rice accessions with second and third PCs, the color was defined by current Admixture analysis. **g** Genome-wide average LD decay estimated from the whole population and each subpopulation

High-quality re-sequencing raw data of 191 accessions derived from the USDA Rice Mini Core (Supplementary Table S1), was retrieved from NCBI SRA database (Accession: PRJNA301661) [52]. Genotyping of the 191 accessions were performed by GATK software. A total of 3,259,478 SNPs was obtained after filtration by minor allele frequencies (≥0.05) and integrity (≥0.4). Imputed SNPs, which were generated by Beagle 5.0 software [53], were used for further analyses. Distribution of these SNPs in the genome is summarized in Table 1 and Fig. 1a, and the overall SNP density in the genome was 114.51 (bp/SNP). The number of SNPs ranged from 212,238 to 375,296 across the twelve rice chromosomes. Chromosome 4 held the minimum marker density with 127.23 (bp/SNP), while chromosome 11 exhibited a maximum marker density with one SNP per 100.40 bp.

**Table 1:** Summary of the SNPs across 12 chromosomes of *Oryza sativa*

Chromosome	Number of SNPs	Length of Chromosome (bp)	Density of SNP (bp/SNP)
1	375,296	43,270,923	115.30
2	301,111	35,937,250	119.35
3	294,312	36,413,819	123.73
4	279,049	35,502,694	127.23
5	253,001	29,958,434	118.41
6	287,238	31,248,787	108.79
7	253,651	29,697,621	117.08
8	261,070	28,443,022	108.95
9	212,238	23,012,720	108.43
10	222,521	23,207,287	104.29
11	289,053	29,021,106	100.40
12	230,938	27,531,856	119.22
Total	3,259,478	373,245,519	114.51

### Population structure and linkage disequilibrium

Admixture analysis divided the 191 accessions into four ancestries, including Indica (63 accessions), Aus (37 accessions), Temperate Japonica (28 accessions), and Tropical Japonica (31 accessions) under the best K model (K = 4) (Fig. 1b), which was determined by the lowest CV (cross-validation error) score (Fig. 1c). Thirty-two accessions are classified as admixture (ADM) since the ratio from each single subpopulation is below 70%.

In order to reduce the amount of calculations, high-quality SNPs (SNPs integrity above 0.8) were selected to construct a maximum likelihood (ML) tree to illustrate the phylogenetic relationship of the 191 rice accessions (Fig. 1d). The population was divided into four subpopulations and the color for each clade was determined according to the Admixture analysis results. The relationship obtained from phylogenetic tree is in line with the Admixture analysis.

Principal component analysis (PCA) was performed based on the 3,259,478 SNPs. Four conceivable subpopulations were separated by PC1, PC2, and PC3. The first three principal components (PCs) explained over 50% of the genetic variation. The first PC separates Indica and Japonica subpopulations (35.70%), the second PC distinguishes the Aus and Indica varieties, while the third PC separates Temperate Japonica and Tropical Japonica varietal groups (Fig. 1e and f). Based on the results from the Admixture analysis, phylogenetic tree and PCA, the population was divided into four subgroups. In addition, the decay of LD with the physical distance between SNPs in all population occurred at 191 kb (*r*^*2*^ = 0.2) (Fig. 1g), which is similar to that of a previous study [54]. Indica subpopulation exhibited the most rapid LD decay and Temperate Japonica showed the most extended LD.

### Correlation of different traits

Correlation analyses between grain ionomics in flooded environment and agronomic traits (Supplementary Fig. S1a), between grain ionomics in unflooded environment and agronomic traits (Supplementary Fig. S1b), and between grain ionomics in flooded versus unflooded growth conditions (Supplementary Fig. S1c) were conducted. The results showed that days to flowering has strong correlation with Rb in flooded (0.53) and unflooded (0.57) (Supplementary Fig. S1a and b) environments. The accumulation of Cd, Mo, and Rb in rice grain in flooded environment and unflooded environment are correlated at *r* = 0.52, 0.81, and 0.60, respectively (Supplementary Fig. S1c).

### Genome-wide association study by univariate GWAS and multivariate GWAS

**Fig. 2:**
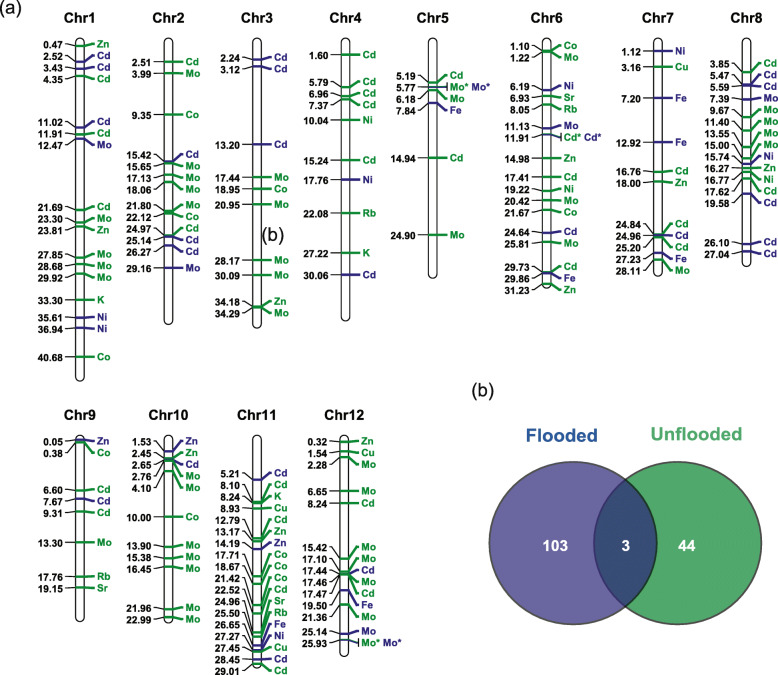
QTLs related to ionomic traits. **a** Distribution of significant QTLs for ionomic traits across the 12 chromosomes of rice under flooded and unflooded environment. Leading SNP was mapped to the chromosome to represent the QTLs’ physical location (Mb). The physical position of each lead SNP was shown on the left side and the corresponding ionomic traits displayed on the right side. QTLs from different growth conditions were distinguished by different colors: green, flooded condition; blue, unflooded condition. An asterisk indicates the locus which was detected from both conditions, **b** The Venn diagram shows the numbers of overlapped loci within or between different conditions

Sixteen grain ionomic traits (As, Ca, Co, Cd, Cu, Fe, K, Mg, Mn, Mo, Ni, P, Rb, S, Sr, and Zn) under flooded and unflooded conditions were the same as reported [17]. Thirteen agronomic traits, including AMYLOSE, AWNTYPE, DAYSFLOWER, HULLCOLOR, HULLCOVER, KERNELLEN, KERNELWID, KERNELRAT, KERNELWT, LODGING, PANICLETYPE, PLANTHT, and PLANTTYPE [18] were shared by Yan as reported [55, 56] and recorded using the methods described by Li et al [57–59]. All these traits were analyzed using two univariate GWAS (GLM and MLM) and two multivariate GWAS (MLMM and FarmCPU) methods to identify QTLs. A total of 106 significant QTLs (*p*-value < 1.53 × 10^− 8^) were detected to be associated with concentrations of 9 ionomic traits (Cd, Co, Cu, K, Mo, Ni, Rb, Sr, and Zn) in rice grain under flooded condition, in which 63, 68, 17, and 44 significant QTLs were identified by GLM, MLM, MLMM, and FarmCPU, respectively (Fig. 2 and Supplementary Fig. S4b). For Cd, twenty-eight significant QTLs were identified. Three of them located near published genes (*CAL1* [14], *OsHMA2* [60], *rgMT* [61]) which have shown to be related to Cd accumulation or resistance. Seven of them were identified in previous mapping studies using univariate methods (Supplementary Table S2). All of the seven QTLs were identified by univariate GWAS methods (GLM and MLM) but only two of the seven were also detected by multivariate methods (MLMM and FarmCPU) in our study. For Co, a total of eleven significant QTLs were detected. Two (one was identified by univariate methods and the other was detected by FarmCPU) of them co-located with previously reported QTLs. Nine of them were new QTLs discovered in the current study, MLMM method discovered 2 significant QTLs and FarmCPU method identified 7 QTLs, respectively. Three QTLs were detected to be significantly associated with K, one of which (only detected by FarmCPU) was also detected in previous studies [62]. For Zn, ten significant QTLs were identified, three of which co-located with previously reported loci [12, 13, 62, 63]. Among them, one significant QTL posited around 18,001,929 bp of Chromosome 7 was detected by both univariate and multivariate methods, which located near reported QTL *qZN-7* [13]. The other two QTLs were detected by FarmCPU method only.

In the unflooded environment, only 47 QTLs were detected to be significantly associated with Cd, Fe, Mo, Ni, and Zn concentration. Specifically, 29, 25, 10, and 20 significant QTLs were identified by GLM, MLM, MLMM, FarmCPU, respectively (Fig. 2 and Supplementary Fig. S4c). Twenty-three identified QTLs were related to Cd, one of which located near *CAL1* gene [14], eight QTLs co-located with previous studies (Supplementary Table S2). Among the eight co-localization QTLs, five were detected by univariate methods and three were identified by multivariate methods. For Fe, seven significant QTLs were identified, two of which were also reported by previous studies [62, 63], and both were detected by FarmCPU only in the current study. We noticed that for the traits that many QTLs were identified using GLM and MLM methods, the numbers of QTLs identified by MLMM and FarmCPU were less as shown in the case of Cd and Mo. When GLM and MLM method failed to identify or only identified a few significant QTLs, QTLs were successfully identified by MLMM and FarmCPU methods as shown in the case of identifying QTLs for Co, Fe, K, and Zn concentration regulation (Supplementary Fig. S2). The QQ-plots in Fig. S2 (c) shows the power of MLMM and FarmCPU, which indicated no evidence for inflation but strong evidence for real effects. In contrast, the QQ-plots of GLM and MLM in Fig. S2 (c) shows the tendency of false positive peaks. This observation was further confirmed when mining the key candidate genes controlling ionomic and agronomic traits as shown in the section below. Interestingly, only 3 of the 106 (ionomic) QTLs identified in flooded growth condition were shared with the QTLs identified in unflooded condition. The three QTLs (QTLs marked with an asterisk on Chromosome 5, 6, and 12; Fig. 2, Supplementary Fig. S2, and Supplementary Table S2) share by both growth condition were associated with Cd and Mo concentration regulation, indicating that the traits of these three QTLs were not impacted by water conditions. Furthermore, several loci were shown to be associated with more than one trait, indicating these QTLs may be pleiotropy. For example, the region around 15.5 Mb on chromosome 2 is associated with Cd and Mo (Fig. 2).

**Fig. 3:**
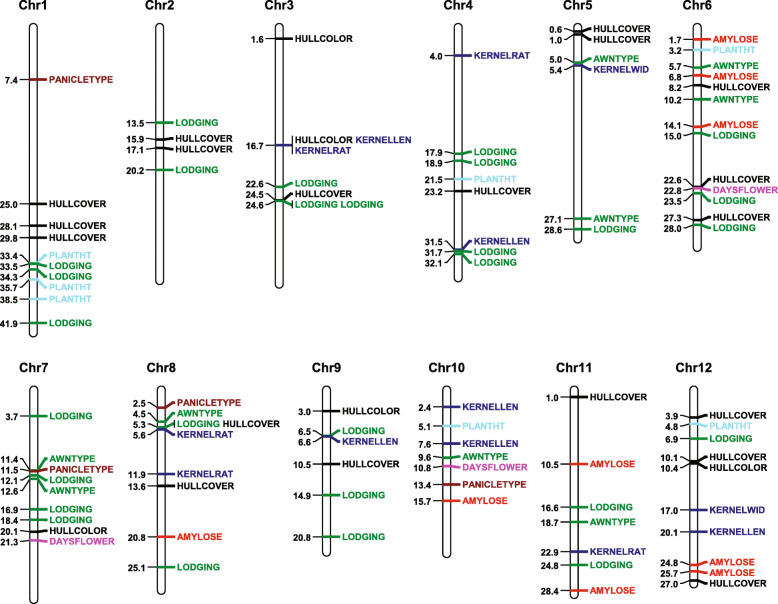
Distribution of significant QTLs for agronomic traits on 12 Chromosomes. Leading SNP was mapped to the chromosome to represent the QTLs’ physical location (Mb). The physical position of each lead SNP was shown on the left side and the corresponding agronomic traits displayed on the right side. QTLs of different type of agronomic traits were distinguished by different colors: red, amylose; blue, grain size (kernel length, kernel width, and kernel rate); black, hull cover and hull color; purple, days to flower; brown, panicle type; green, lodging and awn type

For agronomic traits, a total of 97 significant QTLs (*p*-value < 1.53 × 10^− 8^) were detected for the thirteen agronomic traits described above except for KERNELWT and PLANTTYPE (Fig. 3, Supplementary Table S2). In detail, 39, 16, 29, and 50 significant QTLs were identified by GLM, MLM, MLMM, and FarmCPU, respectively (Supplementary Fig. S4a). *Wax* [64] and *ALK* [65] genes were shown to be significantly associated with amylose content, which is consistent with previous reports. Gain size is a key agronomic trait that strongly linked to yield and quality. Many QTLs have been reported associating with rice grain size, which is decomposed into grain length, width, and thickness (*GS3* [66], *GS5* [67], *GW5* [68], *GW8* [69], *GL7* [70], *TGW6* [71], etc.). In this study, four types of rice grain size-related traits included kernel length (KERNELLEN), kernel width (KERNELWID), kernel rate (KERNELRAT), and kernel weight (KERNELWT) were analyzed. A total of 13 QTLs were detected by univariate and multivariate GWAS methods. Among them, three were detected by univariate (GLM or MLM) GWAS methods and twelve of them were detected by multivariate (MLMM and FarmCPU) GWAS methods. Six, two, and five of the 13 QTLs were found to be associated with KERNELLEN, KERNELWID, and KERNELRAT, respectively. No significant QTL was shown to be associated with KERNELWT. Remarkably, one QTL (Chromosome 3 position 16,733,441) was detected by all the four methods (Supplementary Fig. S3f). The QTL locates on gene *GS3* [66], which is a major gene regulating grain size and organ size. It is worth noting that, five more significant SNPs were identified by FarmCPU and one of them located on chromosome 4 situated nearby the *NAL1* gene, which has been shown to be related to rice yield [72]. For the trait of KERNELWID, two significant QTLs were only detected by MLMM method solely and other methods failed to identify candidate QTLs. One of the identified QTLs positioned around 5,364,561 bp of chromosome 5, which was found approximately 0.56 kb apart from a well-known *GW5* gene controlling rice grain width (Supplementary Fig. S3g) [73]. These results demonstrated the power of GWAS, especially the power of the multivariate (MLMM and FarmCPU) GWAS methods.

### Mining candidate genes of agronomic-related traits

Lodging and Plant height are both related to cell wall properties, which could impact rice yield. Appropriate plant height and the strong stem are required for stable production [74]. A cluster of SNPs around 33.4 Mb on chromosome 1 (Lodging: 33,010,693 to 33,975,764 with leading SNP at 33,469,251; Plant height: 33,181,529 to 33,730,067 with leading SNP at 33,363,796) is shown to be significantly associated with lodging and plant height (Supplementary Fig. S3j and k). Through LD block analysis, we defined a 72.37 kb blocks (33,458,683- 33,531,049) containing 12 genes to be the candidate locus. Among these genes, *OsPME6* (*Os01g0788400*) is annotated as pectin methylesterase 6, which is related to the cell wall modification process. We further conducted blastP analysis with *Arabidopsis thaliana* and found that it shares high homology (E value = 3E-178) with Arabidopsis gene *PME18* (*AT1G11580*) (Supplementary Table S3). The expression of *PME18* increased dramatically under hyper gravity stimulus. It is speculated that pectin esterases induced pectin demethylation of carboxyl groups which increased the rigidity of pectin gel in the cell wall through calcium bridges [75]. Therefore, it is worth to test if *OsPME6* regulates rice lodging and height.

Flowering time is another important trait critical to rice production. Rice is a typical short-day (SD) flowering plant whose flowering is greatly affected by day length. A number of genes [76–79] have been reported to regulate rice flowering-time. In the current study, a total of three QTLs were significantly associated with the flowering time. Two of them were detected by FarmCPU exclusively on Chromosome 7 and 10. The other QTL on Chromosome 6 was detected by all the four different GWAS methods (Supplementary Fig. S3c). The haplotype analysis showed that this region only harbored 2 genes (*OsPLL9* and *OsPLL10*). Among them, *OsPLL9* (*Os06g0583900*) located 7.15 kb away from the leading SNP. This gene is a homolog of pectate lyase gene, which may play crucial roles during rice panicle development [80]. *OsPLL9* is highly expressed in Stamen (one day before flowering), Palea (one day before flowering), and Panicle5 (heading stage) (Supplementary Fig. S5). Thus, *OsPLL9* has the potential to be a candidate gene with a critical role in rice flowering.

### Mining candidate genes of ionomic traits

As a result, 28 and 23 significant QTLs were detected to be associated with Cd concentration in the flooded and unflooded environment, respectively. QTLs near *CAL1* (Chr2:25,190,487-25,191,188) were associated with rice grain Cd accumulation in both flooded (Leading SNP; Chr2: 24,968,588) and unflooded condition (Leading SNP; Chr2: 25,143,071). *CAL1* was annotated as a defensin-like protein, which could regulate Cd accumulation of rice leaves through translocating Cd from cytosol into extracellular spaces, but not rice grains [14]. We then further analyzed the genes around the QTLs and found that there is an ABC transporter (*Os01g0121700*), its phosphorylation level was up-regulated under high Cd treatment (100 μM CdCl_2_·2.5H_2_O) and it has been shown that the transporter reduces the concentration of Cd ^2+^ through transporting PCs-Cd into vacuole [81]. Another QTL (Chr6: 29,733,715) is also showed strongly related to Cd concentration in rice grain. This QTL locates near a known gene *OsHMA2* (about 253 kb away from the leading SNP, but not in LD region), which may decrease rice grain Cd concentration through suppressing the expression level of *OsHMA2* [60]. In addition, significantly associated SNP (Chr11: 29,014,045) posited near *rgMT* gene, which was a metallothionein protein responded to the Cd stress in *E. coli* [61]. Comparing the QTLs detected in this study with previously reported studies, we found that over fifteen QTLs were co-localized with reported loci. The details were shown in Supplementary Table S2. Meanwhile, thirty-two new QTLs were identified.

**Fig. 4:**
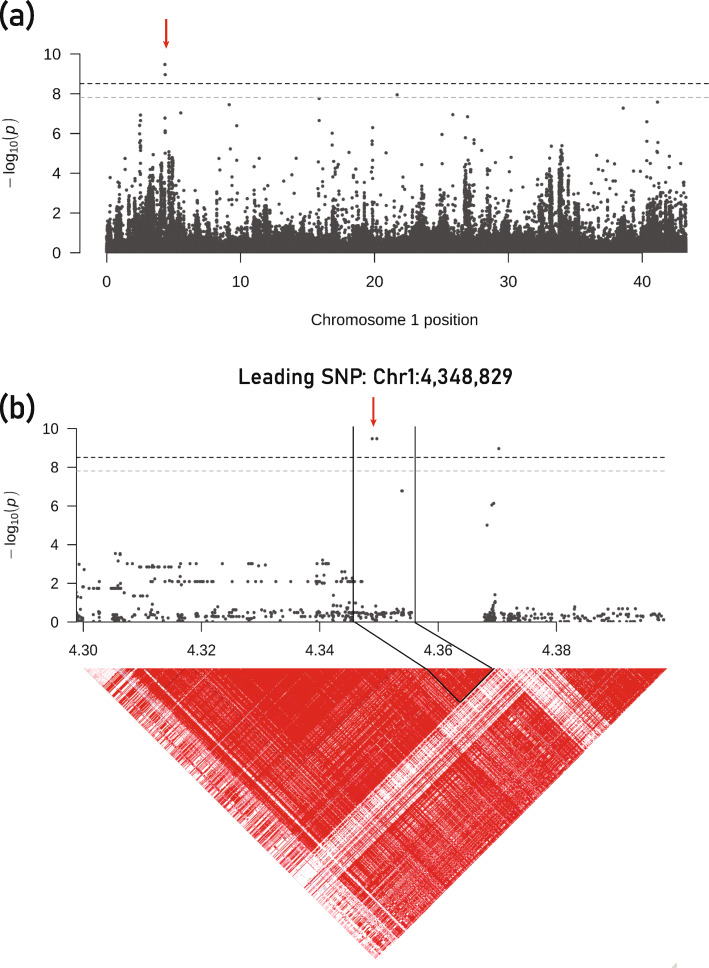
Identification of *OsWRKY102* as a Cadmium concentration QTL in rice grain in flooded condition using MLM method*.* (**a**) Genome-wide association signals on chromosome 1, (**b**) Genome-wide association signals in the region at 4.299–4.399 Mb on chromosome 1 and LD heatmap (bottom)

**Fig. 5:**
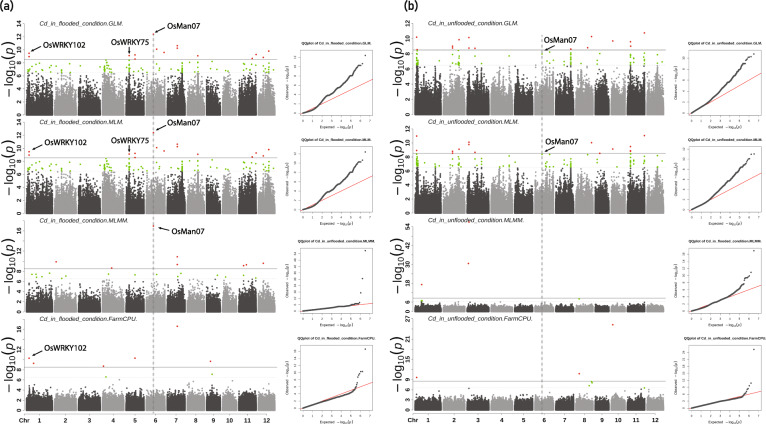
Genome-wide association analysis of Cd concentration with GLM, MLM, MLMM, and FarmCPU methods. **a** in flooded condition and, **b** unflooded condition. Quantile-quantile plot of each model. Black arrows indicated candidate genes. The horizontal dot grey line and green dots indicated the Bonferroni-corrected significance thresholds and SNPs at -log_10_(*p*) = 7.81. The horizontal solid grey line and red dots indicated the Bonferroni-corrected significance thresholds and SNPs at -log10(*p*) = 8.51. The vertical dash grey lines indicate the common QTL detected in flooded and unflooded condition

By applying the procedure mentioned in the methods section, we obtained a list of genes that represent plausible candidates of the causal gene for each of the loci controlling elemental concentrations in rice (Supplementary Table S3 and S5). We select three loci associated with Cd for further investigation with the aim of identifying the causal novel genes. A significant QTL associated with Cd was identified on Chromosome 1 around nucleotide at 4,348,829 bp with *p*-value 3.37E-10 (MLM method). This QTL posited within a 9.97 kb block (Chr1: 4,345,517 - 4,355,489) containing only one candidate gene *OsWRKY102* (*Os01g0182700*) (Fig. 4a and b). BlastP analysis showed that the *OsWRKY102* (*Os01g0182700*) was highly homologous (1.00E-58) to *Arabidopsis WRKY13* (*AT4G39410*) gene (Supplementary Table S4). *WRKY13* activates the expression of gene *PDR8* that encodes a Cd^2+^ extrusion pump, resulting in reduced Cd accumulation [82]. The expression profile from public data showed that *OsWRKY102* was intensively higher expressed in stem comparing to other tissues (Supplementary Fig. S6a). When treated with a high concentration of cadmium, the expression level of *OsWRKY102* increased rapidly in both shoot and root (Supplementary Fig. S6b). Overall, the results suggested that *OsWRKY102* responds at high-level cadmium treatment and regulates cadmium uptake and accumulation in rice. Another QTL (Chromosome 5 posited around 14,941,717) was identified in a flooded environment. Through LD analysis, we defined an 18.65 kb block (Chr5: 14,930,444 - 14,949,090) containing two genes, *Os05g0321600* and *Os05g0321900*. Among them, *Os05g0321900* (*OsWRKY75*) was annotated as DNA-binding WRKY domain-containing protein (Fig. 5a). BlastP analysis found that this gene shared high homology (4E-52) with *WRKY55* (*AT2G40740*) in *Arabidopsis thaliana*, which regulated gold uptake and tolerance. Remarkably, one QTL (Chromosome 6 around position 11,906,590) was identified under both growth environments (Fig. 5a and b). This SNP is a singleton, and we failed to define haplotype blocks for it. For further mining causal gene, we expanded the region as we mentioned in the method section. Finally, we found an important gene *OsMan07* (*Os06g0311600*) was only 24.75 kb away from the leading SNP. This region was reported in previous study [83]*.* BlastP analysis found this gene had a high similarity (6E-108) to *Man3* (*AT3G10890*) gene in *Arabidopsis thaliana* (Supplementary Table S4). Overexpression of *MAN3* enhanced Cd accumulation and tolerance, whereas loss-of-function of *MAN3* led to decreased Cd accumulation and tolerance [84]. All the genes’ expression patterns located in the haplotype region associated with Cd were showed in Supplementary Figure S7. Overall, thirty-two new QTLs were identified in addition to precise identification of the loci reported in previous Cd studies.

*PIP2;6* has been suggested to be involved in arsenic concentration control [85]. Although no SNP with Bonferroni-corrected significant thresholds -log 10(*p*) above 7.81 was discovered, there was an SNP peak with -log(*p*) around 6 on the chromosome 4 near the published gene *PIP2;6* (Supplementary Fig. S2a), suggesting that *PIP2;6* is located near a candidate QTL revealed by GWAS analysis.

### Comparison of univariate and multivariate GWAS methods

Our results demonstrated that there was not a single method that was able to detect all the QTLs while many loci were detected by all methods. The *GS3 gene* was shown to be associated with grain length by all the four tested methods. However, the *GW5 gene* was detected to be related to grain width only by multivariate GWAS. Similarly, the Cu related QTLs in flooded conditions and Fe related QTLs in unflooded condition were also detected by multivariate method only. Interestingly, when a large number of QTLs were identified by the univariate method, the QTLs identified by multivariate method were substantially reduced. For example, over 29 QTLs for Cd were identified by univariate methods in flooded and unflooded environments but only six QTLs were identified by multivariate. Further, it appeared that the multivariate methods were able to pinpoint the location of the QTLs more precisely on the chromosome compared with the univariate methods in many cases. As shown in Supplementary Figure 3f, the peaks identified by univariate method were much broad than the peaks identified by multivariate methods. On the other hand, the univariate methods also identified many loci exclusively. In the case of LODGING, the QTL (candidate gene: *OsPME6*) located on chromosome 1 was only detected by GLM (Supplementary Figure 3j) and this gene was related with cell wall formation biological process, indicating that some of candidate QTLs might be ignored when we pursued lower FDR in multivariate methods.

## Discussion

### The reliability of the GWAS analyses

In this study, many previously reported loci important for agronomic and ionomic traits were rediscovered. The amylose controlling locus *Wax* [86] gene was rediscovered by all four analysis methods and the *ALK* [87] gene was mapped by the two univariate analysis methods. The hull cover genes *OsTCL2* [88] and *OsWOX3* [89] were mapped by the GLM and MLM methods. The kernel length genes *GS3* [66] were mapped by all four methods and the *NAL1* [72] was mapped by the FarmCPU method. The kernel rate gene *GS3* was mapped by GLM, MLMM, and FarmCPU methods and the kernel wide gene *GW5* [73] was mapped by the MLMM method. The two lodging genes (*OsSPL14* [90] *and OsCESA9* [91]) were also successfully mapped by univariate methods. Further, the three known Cd related genes (*CAL1* [14]*, OsHMA2* [60]*, and rgMT* [61]) were mapped by GLM and MLM methods. Since 49 genes/loci with known functions had been successfully remapped, the results confirmed the accuracy of the imputed SNP dataset and the power of mapping QTLs with GWAS. More importantly, these observations also suggested that the 201 new QTLs discovered in this study are worth to be further validated.

### Different GWAS analysis methods possess unique features and complement each other

While many loci were mapped by all of the four tested GWAS methods, each of the four methods identified some of the function known loci exclusively. A kernel length QTL was only identified by FarmCPU method near the yield related gene *NAL1* [72]. The kernel width gene *GW5* [73] on chromosome 5 was only mapped by MLMM method. The lodging gene *OsSPL14* [90] on chromosome 8 was identified by GLM method alone, and the lodging gene *OsCESA9* [91] was only mapped by FarmCPU. There were many genes were mapped by only two or three of the four tested methods. For example, the amylose gene *ALK* [87] on chromosome 6 was mapped by univariate methods GLM and MLM; The kernel rate gene *GS3* [66] on chromosome 3 was mapped by GLM, MLMM, and FarmCPU. These results described above clearly demonstrated that all four methods can be effectively used to perform GWAS analysis and able to identify some of the known loci. However, none of the methods identified all the previously reported loci. Meanwhile, every method successfully mapped some loci while the other three methods failed to identify. Therefore, it is worth to test every method when our goal is to identify as many of related loci as possible. In addition, it is worth noting that the differences within univariate or within multivariate methods are smaller compared to the differences between univariate and multivariate methods. Therefore, our results suggest that it is better to include at least one univariate and one multivariate method in GWAS analyses for the best coverage of the QTLs.

### The relationship between rice grain ionome content and environment

In this study, we conducted the ionomic test of rice grain in flooded and unflooded environment. The results showed the accumulation of most minerals and heavy metals was significantly different in flooded and unflooded environment, indicating the element accumulation in rice grain was affected by water condition, which was coherent with previous studies [92]. However, the content of some minerals and heavy metals was less affected by water conditions. Among them, Mo showed the top consistent accumulation in both flooded and unflooded conditions with correlation efficiency of 0.81, followed by Rb of 0.60, and Cd of 0.52 (Supplementary Fig. S1c). For the identified QTLs, the similar results were obtained. Two Mo QTLs (Chr5:5.77 Mb and Chr12:25.93 Mb) were repeatedly detected in both flooded and unflooded growth conditions. This trend was in agreement with previous study [93], suggesting Mo accumulation in rice was steady and not significantly affected by environment. For the Rb, we only identified significant QTLs in flooded environment, which was not consistent with the correlation study results. For the third-ranked element - Cd, we repeatedly identified one QTL (Chr6: 11.91 Mb) in both flooded and unflooded environments, respectively. Other two Cd concentration related QTLs were detected in a proximate region around 25 Mb on chromosome 2 (24.97 Mb in flooded condition and 25.14 Mb in unflooded condition). The results above suggested that Mo and Cd accumulation in rice grain was barely affected by water conditions while the other elements analyzed in this study were dependent upon the irrigation conditions.

### Potential application of the candidate new genes and significant QTLs in future research and breeding

Forty-nine QTLs or genes with known functions in mineral, heavy metal, or important agronomic trait control were rediscovered in the study. Additionally, 201 new QTL loci with critical roles in regulating these traits were identified. More importantly, multiple candidate genes were identified following programmed and manual sequence analysis of the QTL loci in the mini core collection. Cd and As contamination are a major global concerns for rice production due to widespread contamination resulted from anthropogenic activities and their high toxicity to humans [2]. Cd has an extremely long half-life inside the human body especially in the kidney and liver [94] and inorganic As is a strong carcinogen. We identified 18 QTLs/genes which have been reported to play a role in Cd content control and 32 new QTLs. Moreover, our analyzing results suggested that genes *OsWRKY102* (*Os01g0182700*), *OsWRKY75* (*Os05g0321900*), and *OsMan07* (*Os06g0311600*) are potential candidate genes involved in Cd control in rice. Our results also suggested that the locus with *PIP2;6* gene is probably a QTL involved in As control, thus provided genetic evidence to support prior reports of *PIP2;6*’s potential function in rice, which were based on heterologous expression studies in *Xenopus laevis* oocytes and *Arabidopsis* [85]. The candidate genes involved in heavy metal content regulation are important resources for understanding the underlying mechanism of heavy metal control and agriculture breeding. We found that *OsPME6* has the potential to regulate rice lodging and height and *OsPLL9* has the potential to be a candidate gene with a critical role in rice flowering. Plant height, lodging, and flowering time regulation are critical traits directly related to crop production. Identifying these candidate genes provided new resources for marker development in rice breeding and molecular investigation on the control mechanism of lodging and flower timing. However, it is critical to note that all these candidate genes were identified base on sequence, expression, and homologous analysis of the QTLs, further tests are required to confirm the results before final conclusions.

## Conclusion

In this study, comprehensive GWAS analyses for ionomic and agronomic traits based on 3,259,478 SNPs were performed using two univariate methods and two multivariate methods. Under the criterium *p*-value < 1.53 × 10^− 8^, 106, 47, and 97 QTLs were identified for ionomics in flooded environment, unflooded environment, and agronomic traits, respectively. Under flooded environment, 28, 11, 4, 3, 40, 3, 4, 3, and 10 significant QTLs were shown to be associated with Cd, Co, Cu, K, Mo, Ni, Rb, Sr, and Zn, respectively. In unflooded condition, 23, 7, 7, 7, and 3 significant QTLs were detected to be associated with Cd, Fe, Mo, Ni, and Zn, respectively. In addition, 18, 3, 5, 19, 6, 5, 2, 28, 4, and 7 significant QTLs were tightly associated with amylose concentration, flowering time, hull color, hull cover, kernel length, kernel rate, kernel width, lodging, panicle type, and plant height, respectively. Detailed analysis of the QTLs revealed that 49 of the identified QTLs are co-localized or posited near the genes/QTLs with known functions in the related traits, respectively, and 201 QTLs are newly discovered. Moreover, sequence, expression and homologous analyses of the QTLs suggested that three candidate genes (*OsWRKY102*, *OsWRKY75*, and *OsMan07*) are tightly associated with Cd concentration and *PIP2;6* gene may play role in As regulation in rice. Further, *OsPME6* or nearby gene may regulate plant height and *OsPLL9* or its nearby gene may play a role in flowering time control. Our results showed that each of the four GWAS methods can identify its unique as well as common QTLs and using multiple GWAS methods can complement each other in QTL identification. Using at least one univariate and one multivariate method in GWAS studies is highly recommended for better results. Our comprehensive GWAS analysis of the ionomic and agronomic traits with large scale DNA sequencing data of the USDA mini core collection sets a foundation for further genetic and molecular biology studies on mineral, heavy metal, and agronomic trait regulation.

## Methods

### Plant materials and Phenotyping

Sixteen grain ionomic traits (As, Ca, Co, Cd, Cu, Fe, K, Mg, Mn, Mo, Ni, P, Rb, S, Sr, and Zn) [17] and thirteen agronomic traits (AMYLOSE, AWNTYPE, DAYSFLOWER, HULLCOLOR, HULLCOVER, KERNELLEN, KERNELWID, KERNELRAT, KERNELWT, LODGING, PANICLETYPE, PLANTHT, and PLANTTYPE) [18] of the mini core collection were the same as reported. Diverse rice accessions were grown over 2 years in Beaumont, Texas under both flooded (anaerobic) and unflooded (aerobic, flush irrigated) irrigation schemes for testing ionomics. The planting, field management, and harvest methods were as reported [55–59]. The correlations of the traits were calculated by Pearson’s correlation and visualized with R corrgram package [95]. The details of the samples are listed in Supplementary Table S1.

### Genotyping

In order to obtain high-quality sequencing data, the raw reads were downloaded from NCBI (Accession: PRJNA301661). Through comparing the materials between phenotype and available genotyping data, the overlap of 191 accessions were selected for further analysis. The raw data were firstly filtered by NGS QC Toolkit (v2.3.3) with default settings [96]. Then, the high-quality sequences were mapped to Nipponbare MSU7.0 genomic reference (http://rice.plantbiology.msu.edu/index.shtml, Release 7) with bwa program (version 0.7.17) using default parameters [97]. PCR duplicates were marked by Picard (version 2.18). Then, HaploypeCaller of GATK was used to identify SNPs. The raw SNPs were filtered by PLINK software with parameter ‘--maf 0.05 --geno 0.6 --snps-only’. Genotype imputation was performed for the remaining 3,259,478 SNPs with Beagle 5.0 [97] for further analysis.

### Population structure, genetic analysis, and linkage disequilibrium analysis

The raw SNPs with integrity higher than 0.8 (181,448 SNPs) were extracted for estimating individual ancestries and constructing a phylogenetic tree. A PLINK software tool [98] was used to calculate the potential unlinked SNPs with parameter --indep-pairwise 50 10 0.2. The potentially unlinked SNPs were submitted to ADMIXTURE [99] to assess the population structure with varying K from 2 to 10. Cross-validation error was calculated for each K, and the clustering model with the lowest cross-validation error (K = 4) was selected. Population structure was displayed using online software Pophelper (http://pophelper.com/). Each individual was assigned to one of the four subpopulations based on having ≥70% genetic ancestry derivation, the accessions that had < 70% ancestry from one specific subpopulation were assigned to a fifth group called ‘Admix’. The matrix of pairwise genetic distance was used to construct phylogenetic trees using the software SNPhylo [100] with parameters set to ‘default’. Principal component analysis (PCA) and kinship matrix (K matrix) were performed with 3,259,478 SNPs using default parameter by GAPIT [101]. The decay distance of LD (linkage disequilibrium) in each subpopulation and in the whole mini-core population were determined by software PopLDdecay [102].

### Genome-wide association study (GWAS)

GWAS was performed among 191 rice accessions derived from USDA mini-core collection with 3,259,478 high-quality SNPs. Univariate GWAS methods (GLM and MLM) and multivariate GWAS methods (MLMM and FarmCPU) were employed to evaluate the trait-SNP associations for grain ionomic and agronomic traits using the Genomic Association and Prediction Integrated Tool (GAPIT) [101]. Principal component analysis (PCA) result was used as covariates to correct population structure due to subpopulations in the Mini Core. The genome-wide significant thresholds of the GWAS (*p*-value = 1.53 × 10^− 8^) was determined by 0.05/n (n is the number of markers) [93] and a higher significant threshold was set at 3.06 × 10^− 9^ (0.01/n) [103]. The Manhattan and QQ plots for GWAS were visualized using the R package CMplot (https://github.com/YinLiLin/R-CMplot). Leading SNPs of each significant SNPs cluster (in 200 kb) were selected to display the location of the QTLs.

### Haplotype block estimation

Haplotype blocks containing at least two SNPs were calculated with all imputed SNP using the PLINK software [98] with the following parameters: ‘ --blocks no-pheno-req --blocks-max-kb 2000 --blocks-inform-frac 0.95 --blocks-strong-highci 0.98 --blocks-recomb-highci 0.9’. The haplotypic blocks of each significant SNP were determined by Confidence Intervals described by Gabriel [104]. The LD heatmap was visualized by software Haploview [105]. The annotated genes located in each haplotype block were extracted from RAP-DB (https://rapdb.dna.affrc.go.jp/) (Supplementary Table S5).

### Gene expression data

The gene expression profile across 15 tissues (Endosperm, Callus, Seed, Radicle, Root, Plumule, Stem, Seedling, Shoot, Sheath, Leaf, Panicle, Spikelet, Lemma, and Stamen) was obtained from CREP (Collection of Rice Expression Profiles): http://crep.ncpgr.cn/crep-cgi/home.pl [106]. Gene expression data of rice plants treated with different cadmium concentration [107, 108] was adopted from TENOR (Transcriptome ENcyclopedia Of Rice): https://tenor.dna.affrc.go.jp/.

### Mining causal candidate genes

The QTLs identified by four different GWAS methods provide important clues for understanding the genetic architecture of agronomic and ionomic in rice. To explore candidate genes responsible for each QTLs, we extracted all genes located in the haplotype block of leading SNPs and considering their annotations (Supplementary Table S5). For the leading SNP not posited in the haplotype block, we defined the boundary to within 190 kb of the locus. Besides, functions of homologous gene in *Arabidopsis* and gene expression changes under corresponding stress from published database such as TENOR were also used to narrow down the candidate genes.

## Supplementary information

**Additional file 1: Supplementary Figure 1.** Pearson correlation among ionomics and agronomic traits. (**a**) Ionomics in flooded environment and agronomic traits. (**b**) Ionomics in unflooded environment and agronomic traits. (**c**) Ionomics in flooded environment (with 1 as suffix) and unflooded environment (with 2 as suffix).

**Additional file 2: Supplementary Figure 2.** (**a**). Genome-wide association analysis for As with GLM, MLM, MLMM, and FarmCPU methods (left) in flooded condition. Quantile-quantile plot of each model (right). Red arrow indicates published gene, black arrow indicates candidate gene. The horizontal dot grey line and green dots indicate the Bonferroni-corrected significance thresholds and SNPs at −log10(*P*) = 7.81. The horizontal solid grey line and red dots indicate the Bonferroni-corrected significance thresholds and SNPs at −log10(*P*) = 8.51. (**b**) Genome-wide association analysis for Cd with GLM, MLM, MLMM, and FarmCPU methods (left) in flooded condition. Quantile-quantile plot of each model (right). Black arrows indicate candidate genes. The horizontal dot grey line and green dots indicate the Bonferroni-corrected significance thresholds and SNPs at −log10(*P*) = 7.81. The horizontal solid grey line and red dots indicate the Bonferroni-corrected significance thresholds and SNPs at −log10(*P*) = 8.51. (**c**) Genome-wide association analysis for Cd with GLM, MLM, MLMM, and FarmCPU methods (left) in unflooded condition. Quantile-quantile plot of each model (right). Black arrows indicate candidate genes. The horizontal dot grey line and green dots indicate the Bonferroni-corrected significance thresholds and SNPs at −log10(*P*) = 7.81. The horizontal solid grey line and red dots indicate the Bonferroni-corrected significance thresholds and SNPs at −log10(*P*) = 8.51. (**d**) Genome-wide association analysis for Cu with GLM, MLM, MLMM, and FarmCPU methods (left) in flooded condition. Quantile-quantile plot of each model (right). The horizontal dot grey line and green dots indicate the Bonferroni-corrected significance thresholds and SNPs at −log10(*P*) = 7.81. The horizontal solid grey line and red dots indicate the Bonferroni-corrected significance thresholds and SNPs at −log10(*P*) = 8.51. (**e**) Genome-wide association analysis for Co with GLM, MLM, MLMM, and FarmCPU methods (left) in flooded condition. Quantile-quantile plot of each model (right). The horizontal dot grey line and green dots indicate the Bonferroni-corrected significance thresholds and SNPs at −log10(*P*) = 7.81. The horizontal solid grey line and red dots indicate the Bonferroni-corrected significance thresholds and SNPs at −log10(*P*) = 8.51. (**f**) Genome-wide association analysis for Fe with GLM, MLM, MLMM, and FarmCPU methods (left) in unflooded condition. Quantile-quantile plot of each model (right). The horizontal dot grey line and green dots indicate the Bonferroni-corrected significance thresholds and SNPs at −log10(*P*) = 7.81. The horizontal solid grey line and red dots indicate the Bonferroni -corrected significance thresholds and SNPs at −log10(*P*) = 8.51. (**g**) Genome-wide association analysis for K with GLM, MLM, MLMM, and FarmCPU methods (left) in flooded condition. Quantile-quantile plot of each model (right). The horizontal dot grey line and green dots indicate the Bonferroni-corrected significance thresholds and SNPs at −log10(*P*) = 7.81. The horizontal solid grey line and red dots indicate the Bonferroni-corrected significance thresholds and SNPs at −log10(*P*) = 8.51. (**h**) Genome-wide association analysis for Mo with GLM, MLM, MLMM, and FarmCPU methods (left) in flooded condition. Quantile-quantile plot of each model (right). The horizontal dot grey line and green dots indicate the Bonferroni-corrected significance thresholds and SNPs at −log10(*P*) = 7.81. The horizontal solid grey line and red dots indicate the Bonferroni-corrected significance thresholds and SNPs at −log10(*P*) = 8.51. (**i**) Genome-wide association analysis for Mo with GLM, MLM, MLMM, and FarmCPU methods (left) in unflooded condition. Quantile-quantile plot of each model (right). The horizontal dot grey line and green dots indicate the Bonferroni-corrected significance thresholds and SNPs at −log10(*P*) = 7.81. The horizontal solid grey line and red dots indicate the Bonferroni-corrected significance thresholds and SNPs at −log10(*P*) = 8.51. (**j**) Genome-wide association analysis for Ni with GLM, MLM, MLMM, and FarmCPU methods (left) in flooded condition. Quantile-quantile plot of each model (right). The horizontal dot grey line and green dots indicate the Bonferroni-corrected significance thresholds and SNPs at −log10(*P*) = 7.81. The horizontal solid grey line and red dots indicate the Bonferroni-corrected significance thresholds and SNPs at −log10(*P*) = 8.51. (**k**) Genome-wide association analysis for Ni with GLM, MLM, MLMM, and FarmCPU methods (left) in unflooded condition. Quantile-quantile plot of each model (right). The horizontal dot grey line and green dots indicate the Bonferroni-corrected significance thresholds and SNPs at −log10(*P*) = 7.81. The horizontal solid grey line and red dots indicate the Bonferroni -corrected significance thresholds and SNPs at −log10(*P*) = 8.51. (**l**) Genome-wide association analysis for Rb with GLM, MLM, MLMM, and FarmCPU methods (left) in flooded condition. Quantile-quantile plot of each model (right). The horizontal dot grey line and green dots indicate the Bonferroni-corrected significance thresholds and SNPs at −log10(*P*) = 7.81. The horizontal solid grey line and red dots indicate the Bonferroni-corrected significance thresholds and SNPs at −log10(*P*) = 8.51. (**m**) Genome-wide association analysis for Sr with GLM, MLM, MLMM, and FarmCPU methods (left) in flooded condition. Quantile-quantile plot of each model (right). The horizontal dot grey line and green dots indicate the Bonferroni-corrected significance thresholds and SNPs at −log10(P) = 7.81. The horizontal solid grey line and red dots indicate the Bonferroni-corrected significance thresholds and SNPs at −log10(*P*) = 8.51. (**n**) Genome-wide association analysis for Zn with GLM, MLM, MLMM, and FarmCPU methods (left) in flooded condition. Quantile-quantile plot of each model (right). The horizontal dot grey line and green dots indicate the Bonferroni-corrected significance thresholds and SNPs at −log10(*P*) = 7.81. The horizontal solid grey line and red dots indicate the Bonferroni-corrected significance thresholds and SNPs at −log10(*P*) = 8.51. (**o**) Genome-wide association analysis for Zn with GLM, MLM, MLMM, and FarmCPU methods (left) in unflooded condition. Quantile-quantile plot of each model (right). The horizontal dot grey line and green dots indicate the Bonferroni-corrected significance thresholds and SNPs at −log10(*P*) = 7.81. The horizontal solid grey line and red dots indicate the Bonferroni-corrected significance thresholds and SNPs at −log10(*P*) = 8.51.

**Additional file 3: Supplementary Figure 3. (a)** Genome-wide association analysis for AMYLOSE with GLM, MLM, MLMM, and FarmCPU methods (left). Quantile-quantile plot of each model (right). Red arrows indicate published genes. The horizontal dot grey line and green dots indicate the Bonferroni-corrected significance thresholds and SNPs at −log10(*P*) = 7.81. The horizontal solid grey line and red dots indicate the Bonferroni-corrected significance thresholds and SNPs at −log10(*P*) = 8.51. **(b)** Genome-wide association analysis for AWNTYPE with GLM, MLM, MLMM, and FarmCPU methods (left). Quantile-quantile plot of each model (right). The horizontal dot grey line and green dots indicate the Bonferroni-corrected significance thresholds and SNPs at −log10(*P*) = 7.81. The horizontal solid grey line and red dots indicate the Bonferroni-corrected significance thresholds and SNPs at −log10(*P*) = 8.51. **(c)** Genome-wide association analysis for DAYSFLOWER with GLM, MLM, MLMM, and FarmCPU methods (left). Quantile-quantile plot of each model (right). Black arrow indicates candidate gene. The horizontal dot grey line and green dots indicate the Bonferroni-corrected significance thresholds and SNPs at −log10(*P*) = 7.81. The horizontal solid grey line and red dots indicate the Bonferroni-corrected significance thresholds and SNPs at −log10(*P*) = 8.51. **(d)** Genome-wide association analysis for HULLCOLOR with GLM, MLM, MLMM, and FarmCPU methods (left). Quantile-quantile plot of each model (right). The horizontal dot grey line and green dots indicate the Bonferroni-corrected significance thresholds and SNPs at −log10(*P*) = 7.81. The horizontal solid grey line and red dots indicate the Bonferroni-corrected significance thresholds and SNPs at −log10(*P*) = 8.51. **(e)** Genome-wide association analysis for HULLCOVER with GLM, MLM, MLMM, and FarmCPU methods (left). Quantile-quantile plot of each model (right). The horizontal dot grey line and green dots indicate the Bonferroni-corrected significance thresholds and SNPs at −log10(*P*) = 7.81. The horizontal solid grey line and red dots indicate the Bonferroni-corrected significance thresholds and SNPs at −log10(*P*) = 8.51. **(f)** Genome-wide association analysis for KERNELLEN with GLM, MLM, MLMM, and FarmCPU methods (left). Quantile-quantile plot of each model (right). Red arrow indicates published gene. The horizontal dot grey line and green dots indicate the Bonferroni-corrected significance thresholds and SNPs at −log10(*P*) = 7.81. The horizontal solid grey line and red dots indicate the Bonferroni-corrected significance thresholds and SNPs at −log10(*P*) = 8.51. **(g)** Genome-wide association analysis for KERNELWID with GLM, MLM, MLMM, and FarmCPU methods (left). Quantile-quantile plot of each model (right). Red arrow indicates published gene. The horizontal dot grey line and green dots indicate the Bonferroni-corrected significance thresholds and SNPs at −log10(*P*) = 7.81. The horizontal solid grey line and red dots indicate the Bonferroni-corrected significance thresholds and SNPs at −log10(*P*) = 8.51. **(h)** Genome-wide association analysis for KERNELRAT with GLM, MLM, MLMM, and FarmCPU methods (left). Quantile-quantile plot of each model (right). Red arrow indicates published gene. The horizontal dot grey line and green dots indicate the Bonferroni-corrected significance thresholds and SNPs at −log10(*P*) = 7.81. The horizontal solid grey line and red dots indicate the Bonferroni-corrected significance thresholds and SNPs at −log10(*P*) = 8.51. **(i)** Genome-wide association analysis for KERNELWTB with GLM, MLM, MLMM, and FarmCPU methods (left). Quantile-quantile plot of each model (right). The horizontal dot grey line and green dots indicate the Bonferroni-corrected significance thresholds and SNPs at −log10(*P*) = 7.81. The horizontal solid grey line and red dots indicate the Bonferroni-corrected significance thresholds and SNPs at −log10(*P*) = 8.51. **(j)** Genome-wide association analysis for LODGING with GLM, MLM, MLMM, and FarmCPU methods (left). Quantile-quantile plot of each model (right). Black arrow indicates candidate gene. The horizontal dot grey line and green dots indicate the Bonferroni-corrected significance thresholds and SNPs at −log10(*P*) = 7.81. The horizontal solid grey line and red dots indicate the Bonferroni-corrected significance thresholds and SNPs at −log10(*P*) = 8.51. **(k)** Genome-wide association analysis for PLANTHT with GLM, MLM, MLMM, and FarmCPU methods (left). Quantile-quantile plot of each model (right). Black arrow indicates candidate gene. The horizontal dot grey line and green dots indicate the Bonferroni-corrected significance thresholds and SNPs at −log10(*P*) = 7.81. The horizontal solid grey line and red dots indicate the Bonferroni-corrected significance thresholds and SNPs at −log10(*P*) = 8.51.

**Additional file 4: Supplementary Figure 4.** The number of QTLs identified by four different methods. (a) Agronomic traits; (b) Ionomic traits in flooded environment; (c) Ionomic traits in unflooded environment. Different color represents different methods; blue, GLM; yellow, MLM; green, MLMM; red, FarmCPU. Venn diagrams were visualized by online software Venny 2.1: http://bioinfogp.cnb.csic.es/tools/venny/.

**Additional file 5: Supplementary Figure 5.** Heat map of the putative candidate gene expression patterns (log2-transformed) in 15 tissues in Minghui 63, Shanyou 63, and Zhenshan 97 varieties. Darkblue indicates high expression, white indicates low expression, and grey indicates NA.

**Additional file 6: Supplementary Figure 6.** (**a**) The expression pattern of a candidate gene ( OsWRK102) in different tissues from public data. (**b**) The candidate gene expression profile in different treatment (High Cd [(50 μM CdSO_4_)]), Low Cd [(1 μM CdSO_4_)], and Very Low Cd [(0.2 μM CdSO_4_)] in Shoot and Root.

**Additional file 7: Supplementary Figure 7.** Heat map of the genes’ (located in the blocks) expression patterns under cadmium stress. Red indicates high expression, and blue indicates low expression. Genes with red color are candidate genes in the current study.

**Additional file 8: Supplementary Table 1.** The sample list and structure information of *O. sativa* accessions.

**Additional file 9: Supplementary Table 2.** The QTLs associated with agronomic traits, ionomic in flooded and unflooded conditions with four different methods.

**Additional file 10: Supplementary Table 3.** List of gene orthologous with *Arabidopsis thaliana*.

**Additional file 11: Supplementary Table 4.** The distribution of LD blocks in the 12 chromosomes of rice.

**Additional file 12: Supplementary Table 5.** List of candidate genes located in the LD blocks for agronomic, ionomic in flooded, and ionomic in unflooded traits.

## Data Availability

The genotype datasets analyzed during the current study are available in the NCBI SRA database (Accession: PRJNA301661), the phenotype traits analyzed are available in published article “Worldwide Genetic Diversity for Mineral Element Concentrations in Rice Grain” and website https://npgsweb.ars-grin.gov/gringlobal/descriptors.aspx

## Abbreviations

ADM: Admixture
AMYLOSE: Amylose
As: Arsenic
ATSDR: Agency for Toxic Substances and Disease Registry
AWNTYPE: Awn type
Cd: Cadmium
CREP: Collection of Rice Expression Profiles
DAYSFLOWER: Flowering time
FarmCPU: Fixed and random model Circulating Probability Unification
FASTmrEMMA: Fast multi-locus random-SNP-effect efficient mixed model analysis
FASTmrMLM: Fast mrMLM
FEM: Fixed Effect Model
GAPIT: Genomic Association and Prediction Integrated Tool
GPWAS: Genome-Phenome Wide Association Study
GWAS: Genome-wide-association-study
HULLCOLOR: Hull color
HULLCOVER: Hull cover
ISIS EM-BLASSO: Iterative modified-sure independence screening expectation-maximization-Bayesian least absolute shrinkage and selection operator
KERNELLEN: Kernel length
KERNELRAT: Kernel rate
KERNELWID: Kernel width
KERNELWT: Kernel weight
LD: Linkage disequilibrium
LODGING: Lodging
ML: Maximum likelihood
MLMM: Multi-locus mixed-model
Mn: Manganese
mrMLM: Multi-locus random-SNP-effect MLM
Ni: Nickel
NSGC: National Small Grains Collection
PANICLETYPE: Panicle type
PCA: Principal component analysis
PCs: Principal components
pKWmEB: Integration of Kruskal-Wallis test with empirical Bayes
PLANTHT: Plant height
PLANTTYPE: Plant type
pLARmEB: Polygenic-background-control-based least angle regression plus empirical Bayes
QTL: Quantitative Trait Locus
SD: Short-day
TENOR: Transcriptome ENcyclopedia Of Rice
