# Supplementary material for: Genome-wide association studies of ionomic and agronomic traits in USDA mini core collection of rice and comparative analyses of different mapping methods
Source: BMC Plant Biol. 2020 Sep 24;20:441. doi: 10.1186/s12870-020-02603-0 (PMC7513512; doi:10.1186/s12870-020-02603-0)
Supplement: Supplementary file 1 — Additional file 1: Supplementary Figure 1. Pearson correlation among ionomics and agronomic traits. (a) Ionomics in flooded environment and agronomic traits. (b) Ionomics in unflooded environment and agronomic traits. (c) Ionomics in flooded environment (with 1 as suffix) and unflooded environment (with 2 as suffix). [file 12870_2020_2603_MOESM1_ESM.pdf]

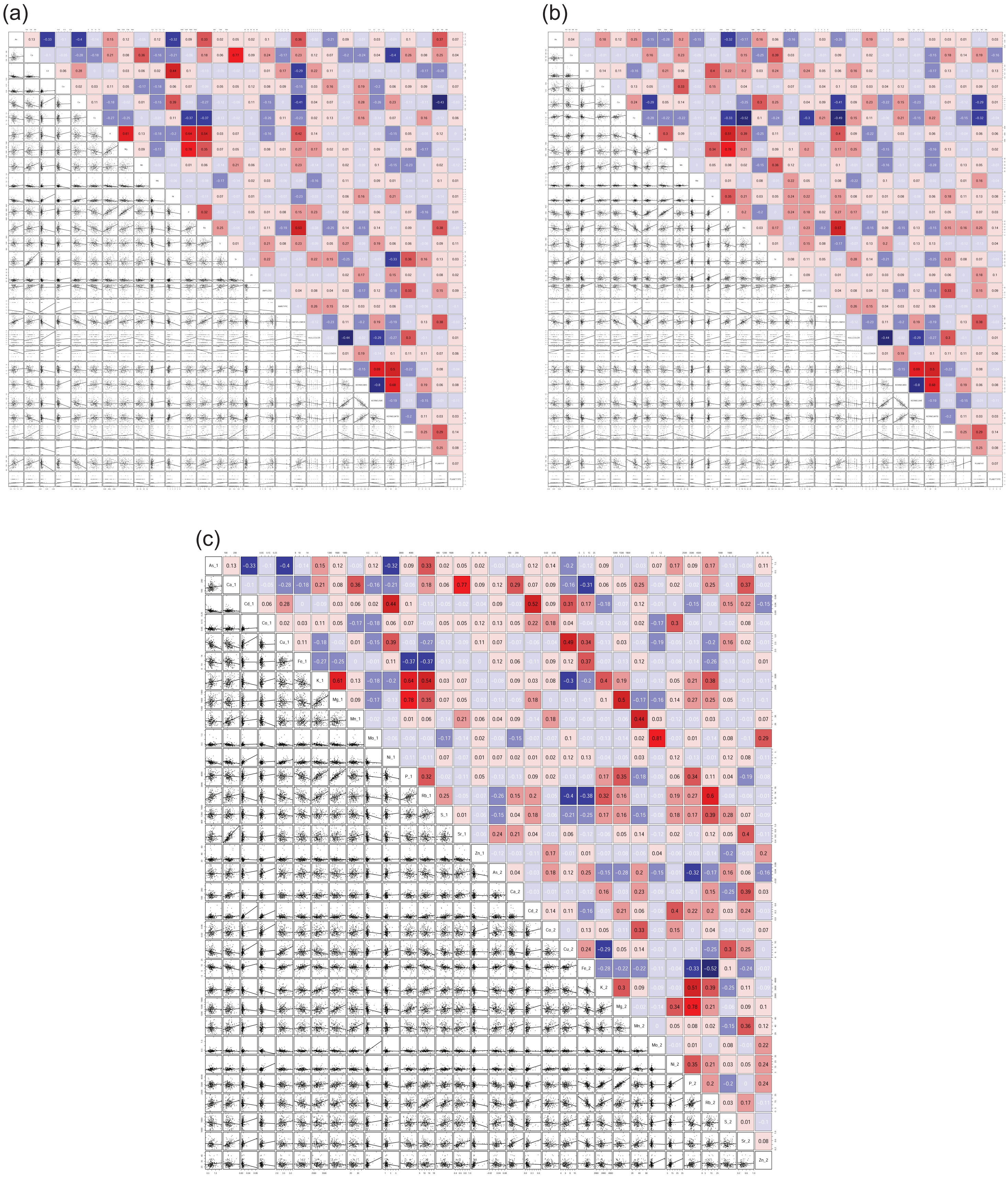

Supplementary Figure 1 Pearson correlation among ionomics and agronomic traits. **(a)** Ionomics in flooded environment and agronomic traits. **(b)** Ionomics in unflooded environment and agronomic traits. **(c)** Ionomics in flooded environment (with 1 as suffix) and unflooded environment (with 2 as suffix).
