# Supplementary material for: Genome-wide association studies of ionomic and agronomic traits in USDA mini core collection of rice and comparative analyses of different mapping methods
Source: BMC Plant Biol. 2020 Sep 24;20:441. doi: 10.1186/s12870-020-02603-0 (PMC7513512; doi:10.1186/s12870-020-02603-0)
Supplement: Supplementary file 2 — Additional file 2: Supplementary Figure 2. (a). Genome-wide association analysis for As with GLM, MLM, MLMM, and FarmCPU methods (left) in flooded condition. Quantile-quantile plot of each model (right). Red arrow indicates published gene, black arrow indicates candidate gene. The horizontal dot grey line and green dots indicate the Bonferroni-corrected significance thresholds and SNPs at −log10(P) = 7.81. The horizontal solid grey line and red dots indicate the Bonferroni-corrected significance thresholds and SNPs at −log10(P) = 8.51. (b) Genome-wide association analysis for Cd with GLM, MLM, MLMM, and FarmCPU methods (left) in flooded condition. Quantile-quantile plot of each model (right). Black arrows indicate candidate genes. The horizontal dot grey line and green dots indicate the Bonferroni-corrected significance thresholds and SNPs at −log10(P) = 7.81. The horizontal solid grey line and red dots indicate the Bonferroni-corrected significance thresholds and SNPs at −log10(P) = 8.51. (c) Genome-wide association analysis for Cd with GLM, MLM, MLMM, and FarmCPU methods (left) in unflooded condition. Quantile-quantile plot of each model (right). Black arrows indicate candidate genes. The horizontal dot grey line and green dots indicate the Bonferroni-corrected significance thresholds and SNPs at −log10(P) = 7.81. The horizontal solid grey line and red dots indicate the Bonferroni-corrected significance thresholds and SNPs at −log10(P) = 8.51. (d) Genome-wide association analysis for Cu with GLM, MLM, MLMM, and FarmCPU methods (left) in flooded condition. Quantile-quantile plot of each model (right). The horizontal dot grey line and green dots indicate the Bonferroni-corrected significance thresholds and SNPs at −log10(P) = 7.81. The horizontal solid grey line and red dots indicate the Bonferroni-corrected significance thresholds and SNPs at −log10(P) = 8.51. (e) Genome-wide association analysis for Co with GLM, MLM, MLMM, and FarmCPU methods (left) in flooded con [file 12870_2020_2603_MOESM2_ESM.pdf]

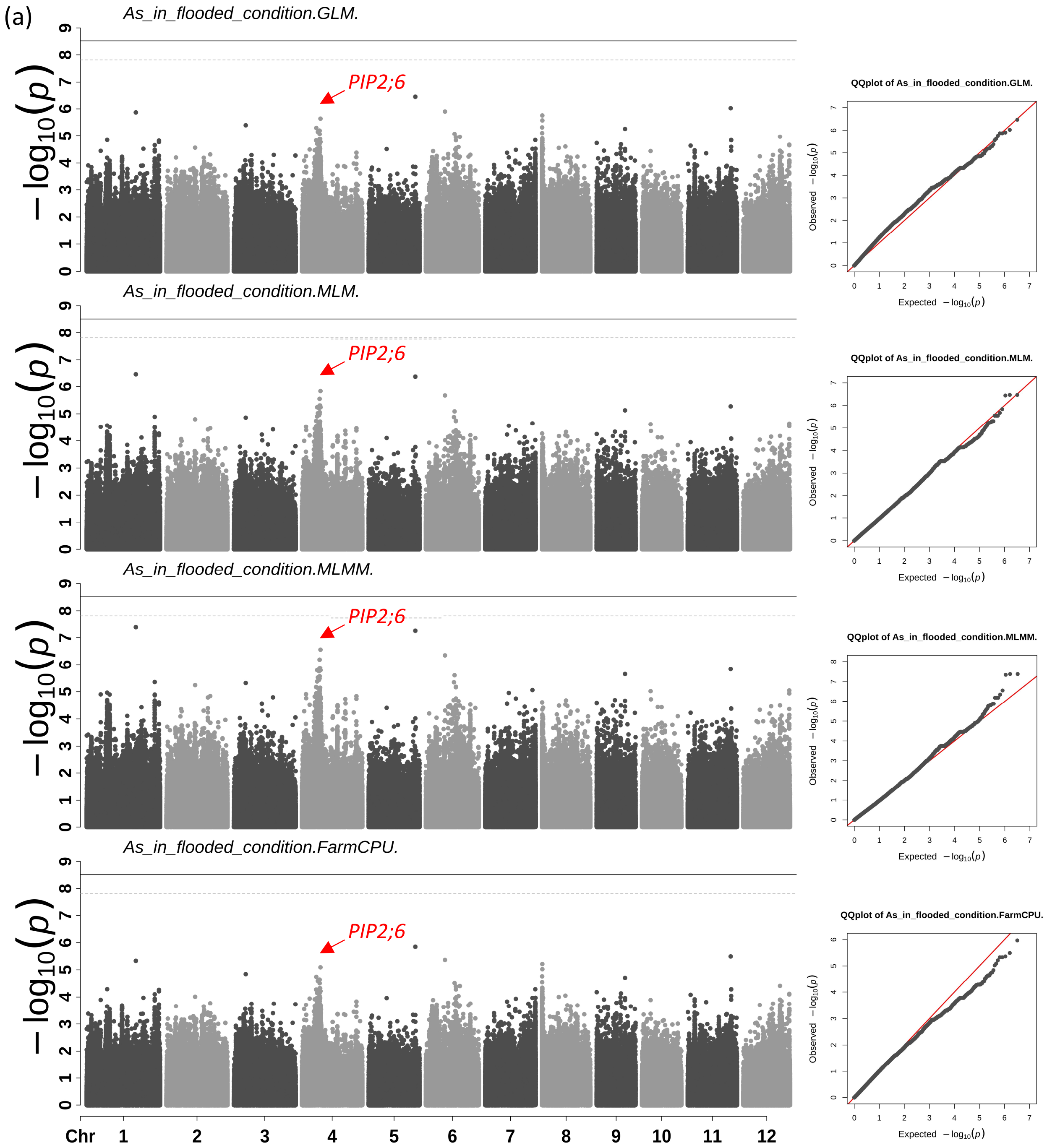


**Supplementary Figure 2 (a)** Genome-wide association analysis for As with GLM, MLM, MLMM, and FarmCPU methods (left) in flooded condition. Quantile-quantile plot of each model (right). Red arrow indicates published gene, black arrow indicates candidate gene. The horizontal dot grey line and green dots indicate the Bonferroni-corrected significance thresholds and SNPs at −log10(*P*) = 7.81. The horizontal solid grey line and red dots indicate the Bonferroni-corrected significance thresholds and SNPs at −log10(*P*) = 8.51.

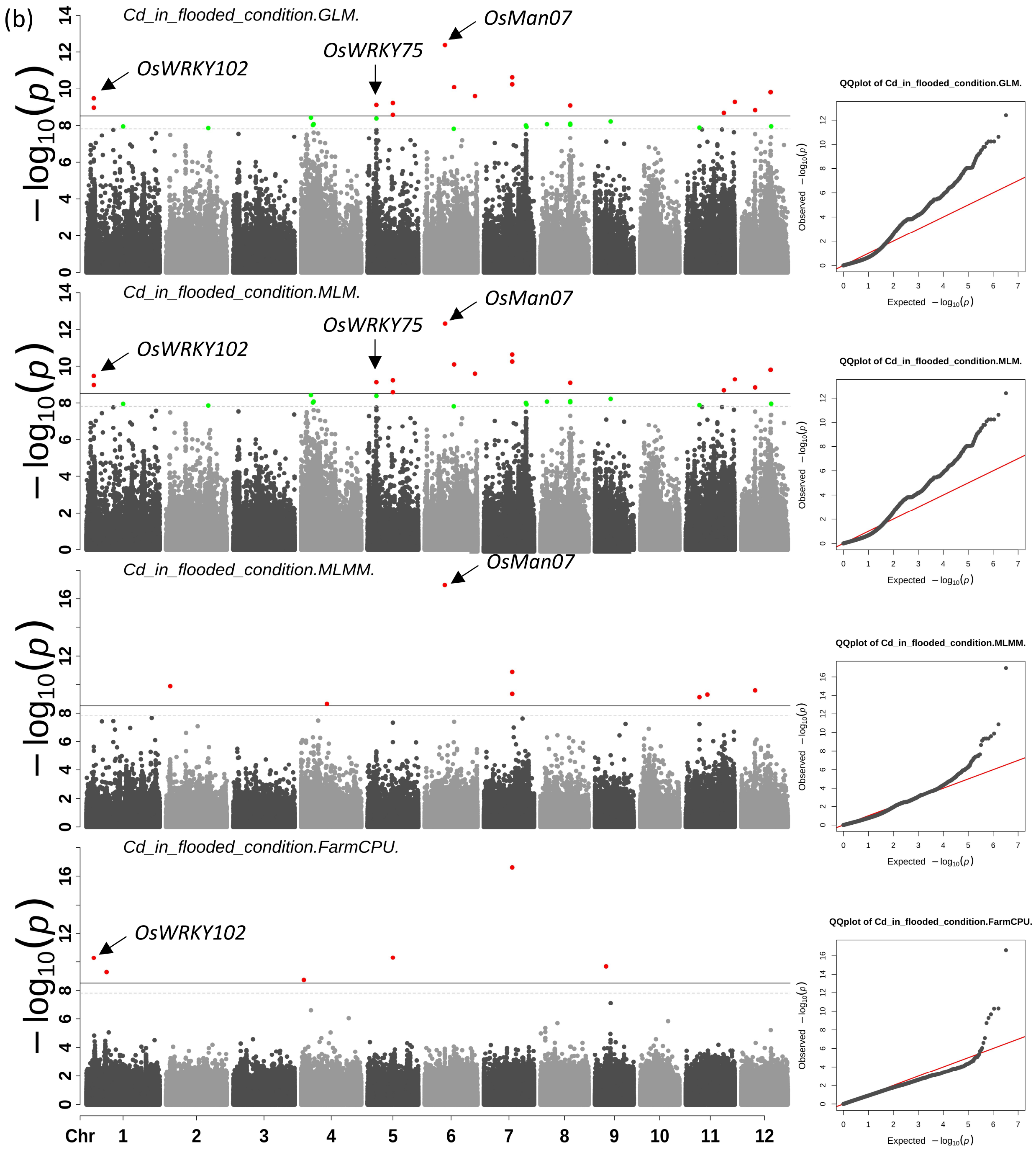


**Supplementary Figure 2 (b)** Genome-wide association analysis for Cd with GLM, MLM, MLMM, and FarmCPU methods (left) in flooded condition. Quantile-quantile plot of each model (right). Black arrows indicate candidate genes. The horizontal dot grey line and green dots indicate the Bonferroni-corrected significance thresholds and SNPs at −log10(*P*) = 7.81. The horizontal solid grey line and red dots indicate the Bonferroni-corrected significance thresholds and SNPs at −log10(*P*) = 8.51.

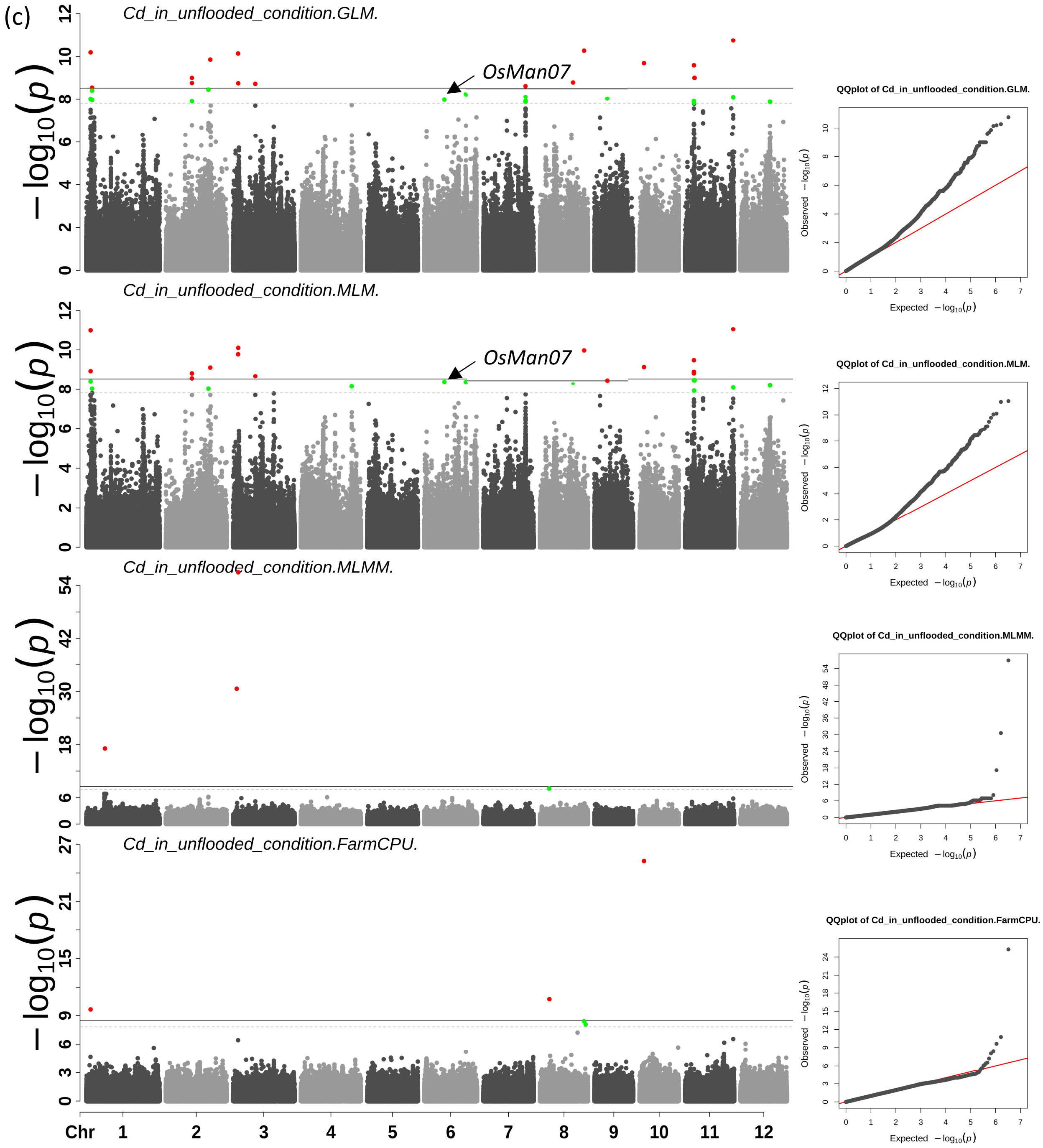


**Supplementary Figure 2 (c)** Genome-wide association analysis for Cd with GLM, MLM, MLMM, and FarmCPU methods (left) in unflooded condition. Quantile-quantile plot of each model (right). Black arrows indicate candidate genes. The horizontal dot grey line and green dots indicate the Bonferroni-corrected significance thresholds and SNPs at −log10(*P*) = 7.81. The horizontal solid grey line and red dots indicate the Bonferroni-corrected significance thresholds and SNPs at −log10(*P*) = 8.51.

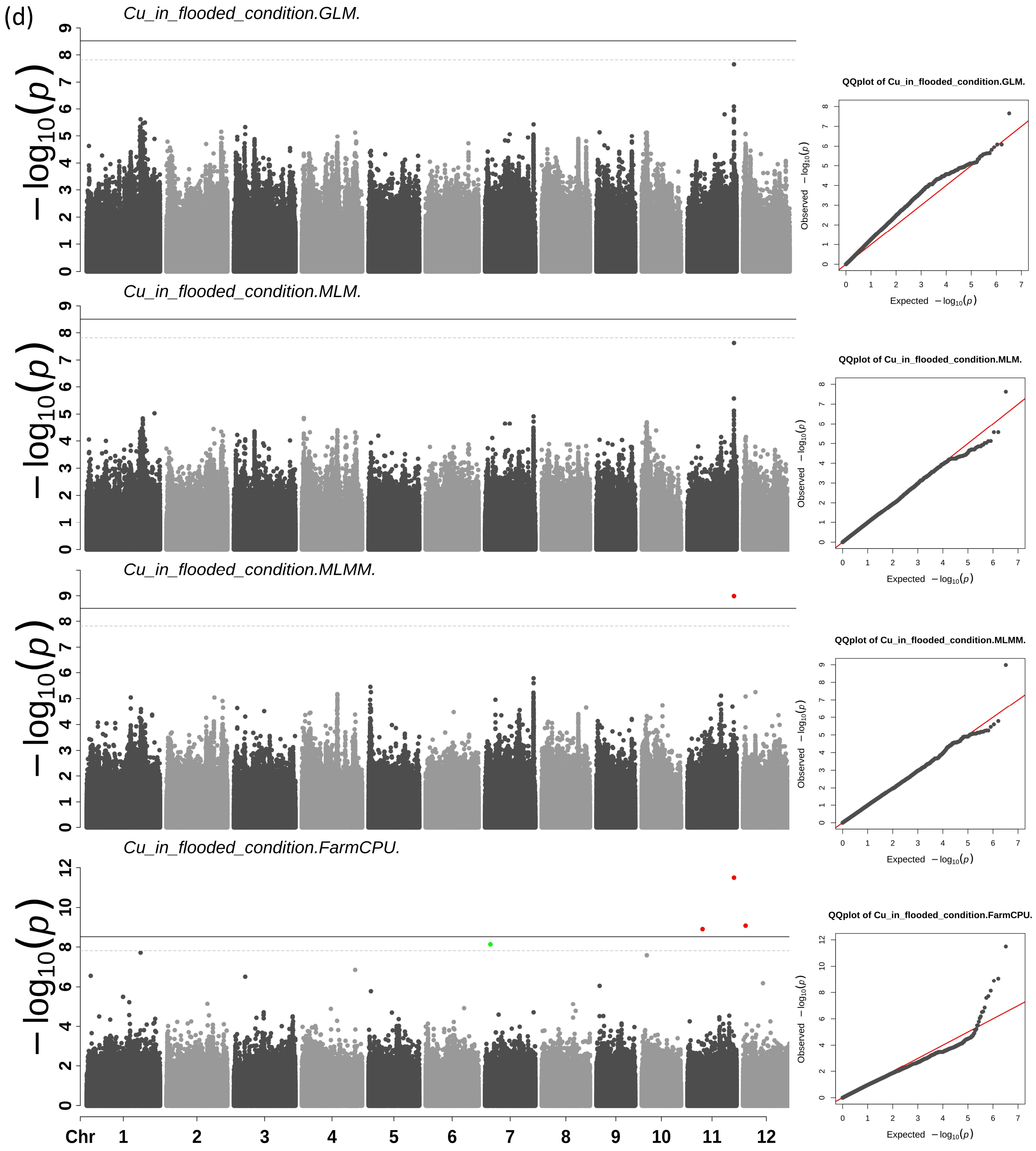


**Supplementary Figure 2 (d)** Genome-wide association analysis for Cu with GLM, MLM, MLMM, and FarmCPU methods (left) in flooded condition. Quantile-quantile plot of each model (right). The horizontal dot grey line and green dots indicate the Bonferroni-corrected significance thresholds and SNPs at −log10(*P*) = 7.81. The horizontal solid grey line and red dots indicate the Bonferroni-corrected significance thresholds and SNPs at −log10(*P*) = 8.51.

(e)

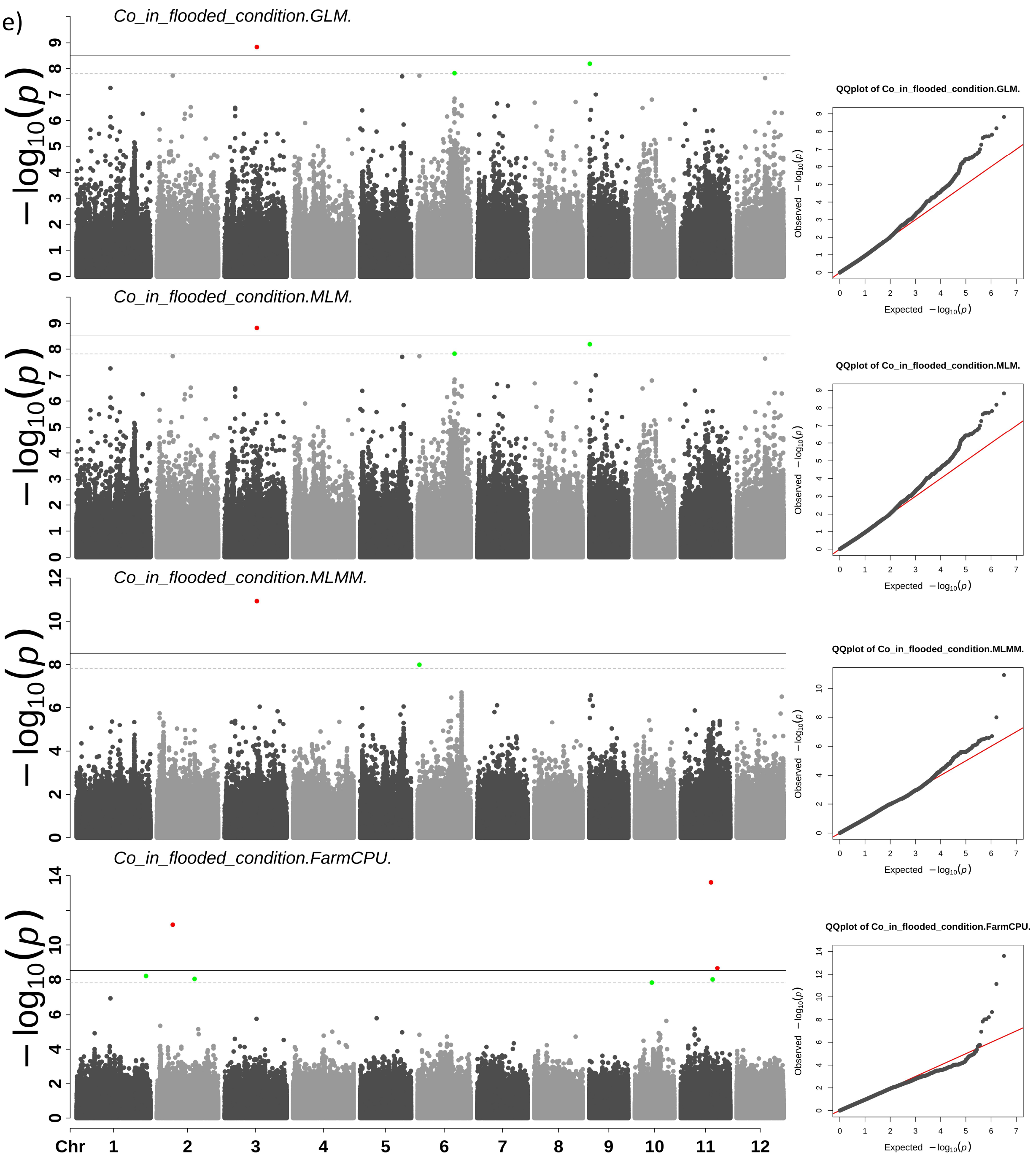


**Supplementary Figure 2 (e)** Genome-wide association analysis for Co with GLM, MLM, MLMM, and FarmCPU methods (left) in flooded condition. Quantile-quantile plot of each model (right). The horizontal dot grey line and green dots indicate the Bonferroni-corrected significance thresholds and SNPs at −log10(*P*) = 7.81. The horizontal solid grey line and red dots indicate the Bonferroni-corrected significance thresholds and SNPs at −log10(*P*) = 8.51.

(f)

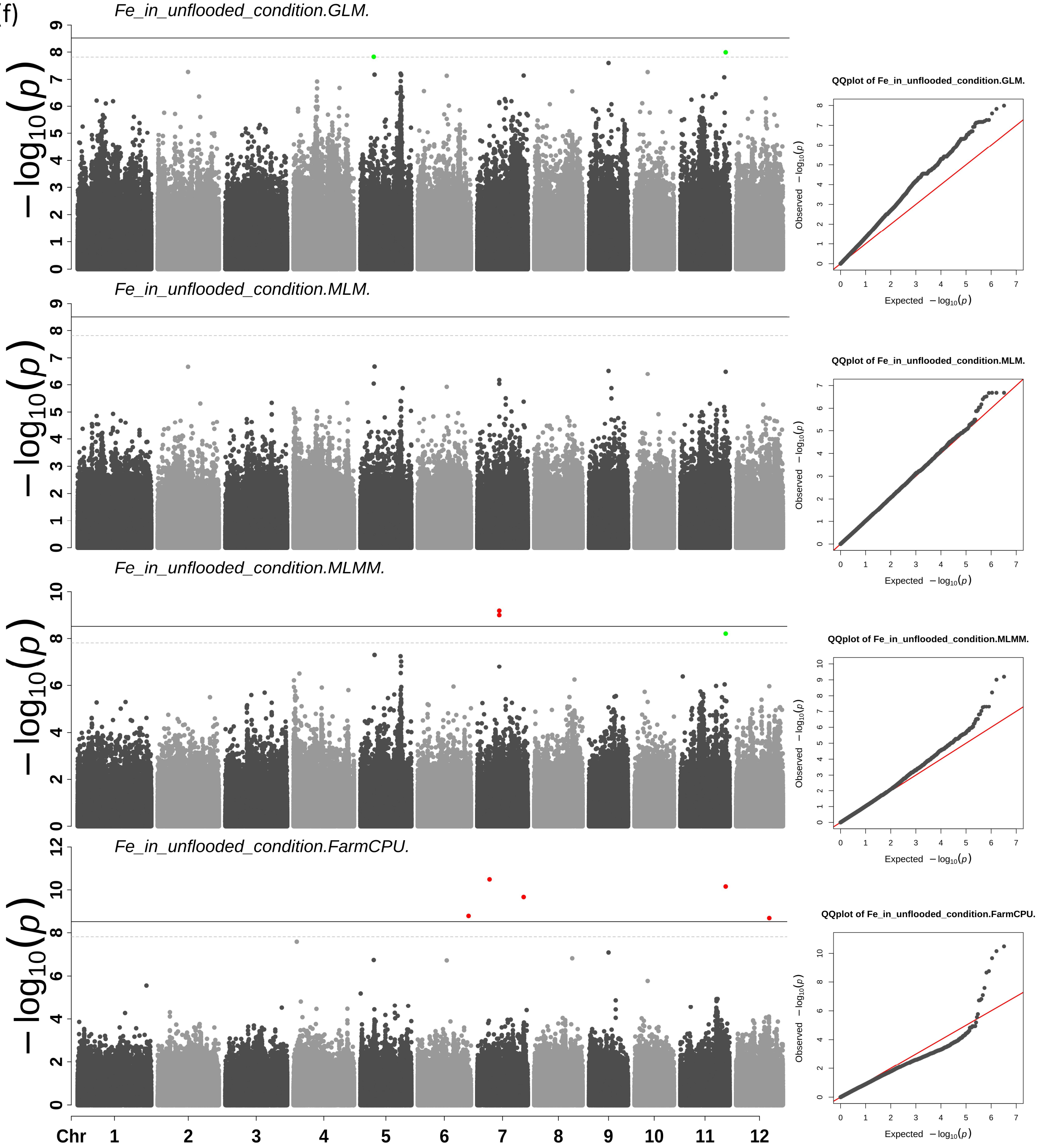


**Supplementary Figure 2 (f)** Genome-wide association analysis for Fe with GLM, MLM, MLMM, and FarmCPU methods (left) in unflooded condition. Quantile-quantile plot of each model (right). The horizontal dot grey line and green dots indicate the Bonferroni-corrected significance thresholds and SNPs at −log10(*P*) = 7.81. The horizontal solid grey line and red dots indicate the Bonferroni-corrected significance thresholds and SNPs at −log10(*P*) = 8.51.

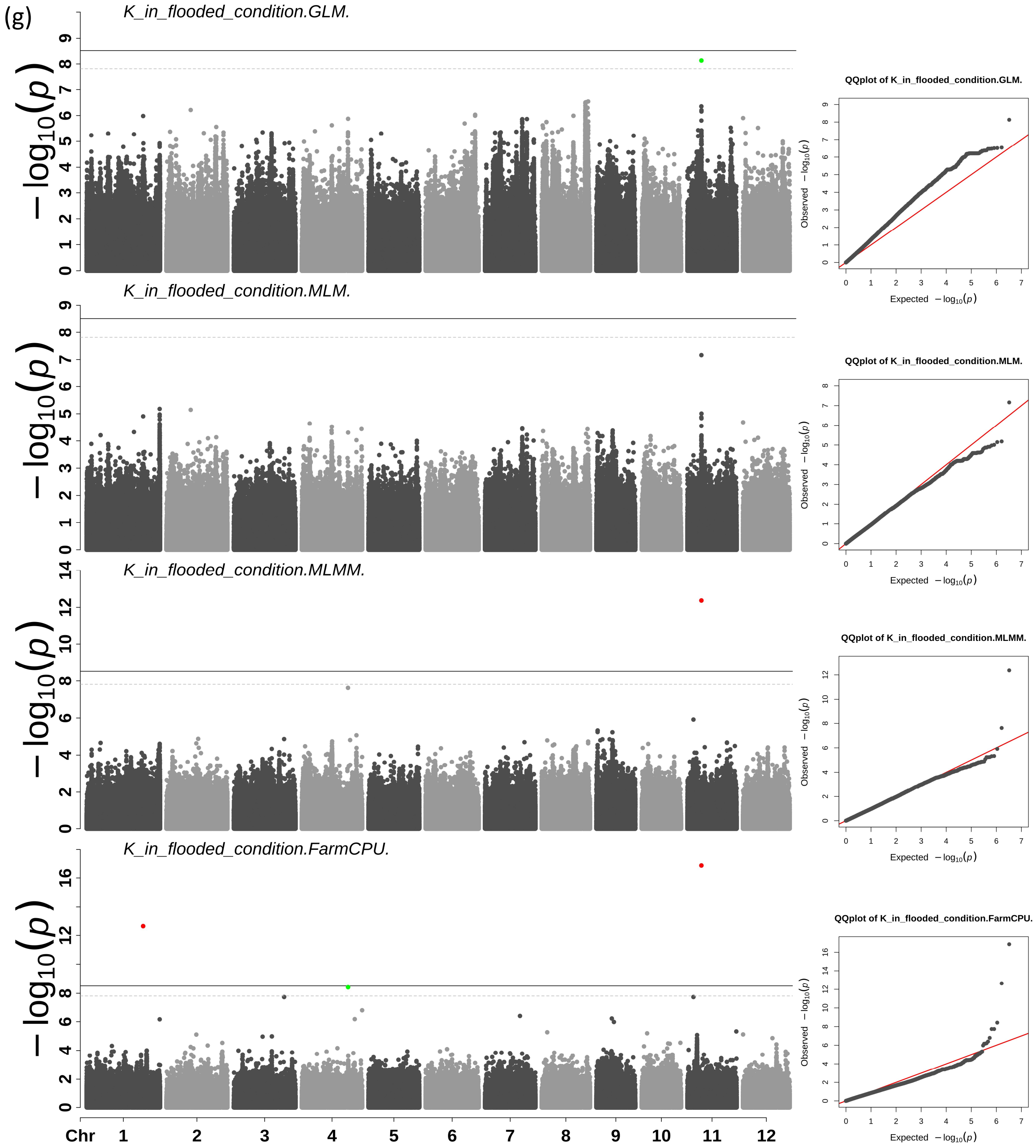


**Supplementary Figure 2 (g)** Genome-wide association analysis for K with GLM, MLM, MLMM, and FarmCPU methods (left) in flooded condition. Quantile-quantile plot of each model (right). The horizontal dot grey line and green dots indicate the Bonferroni-corrected significance thresholds and SNPs at −log10(*P*) = 7.81. The horizontal solid grey line and red dots indicate the Bonferroni-corrected significance thresholds and SNPs at −log10(*P*) = 8.51.

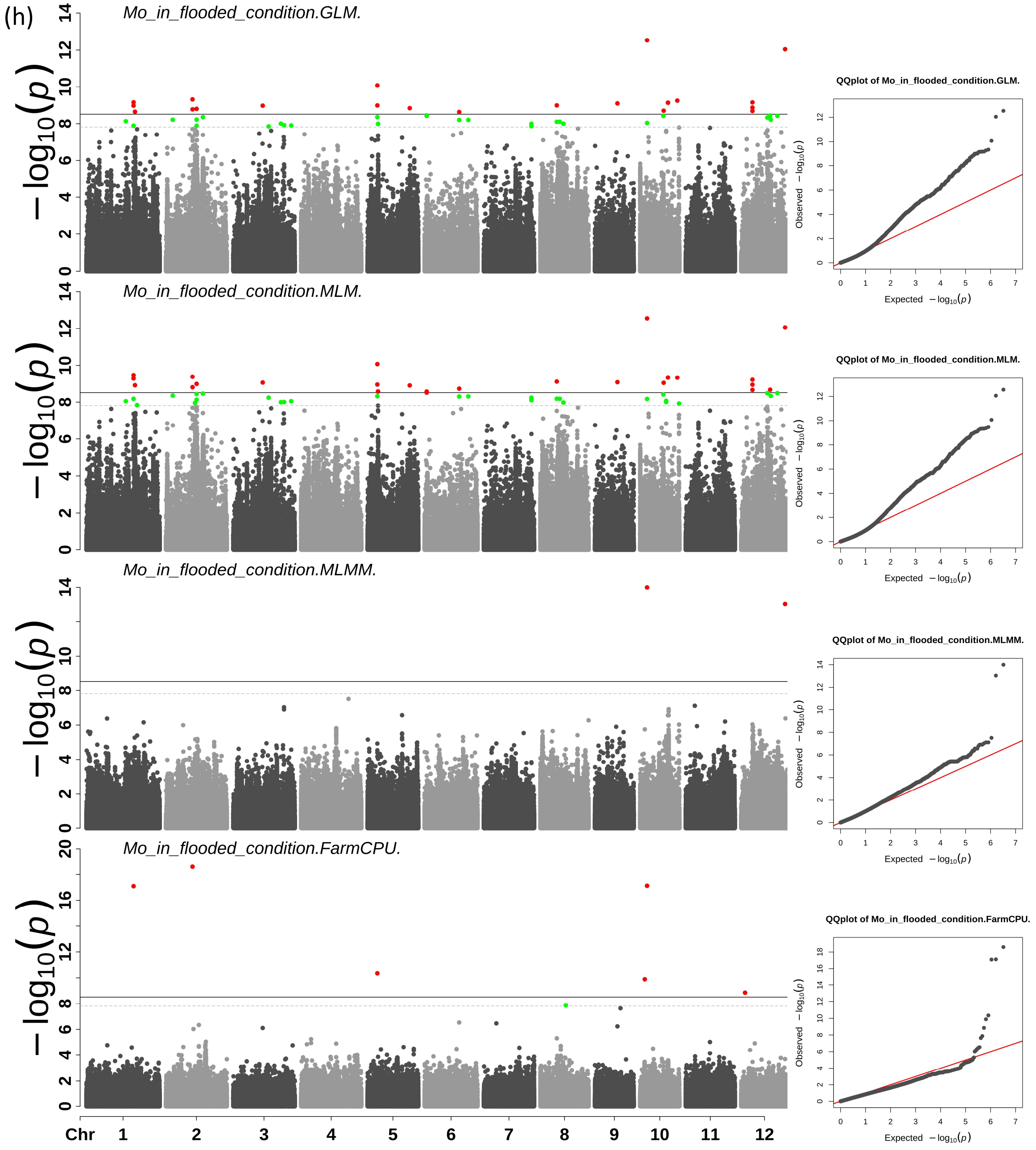


**Supplementary Figure 2 (h)** Genome-wide association analysis for Mo with GLM, MLM, MLMM, and FarmCPU methods (left) in flooded condition. Quantile-quantile plot of each model (right). The horizontal dot grey line and green dots indicate the Bonferroni-corrected significance thresholds and SNPs at −log10(*P*) = 7.81. The horizontal solid grey line and red dots indicate the Bonferroni-corrected significance thresholds and SNPs at −log10(*P*) = 8.51.

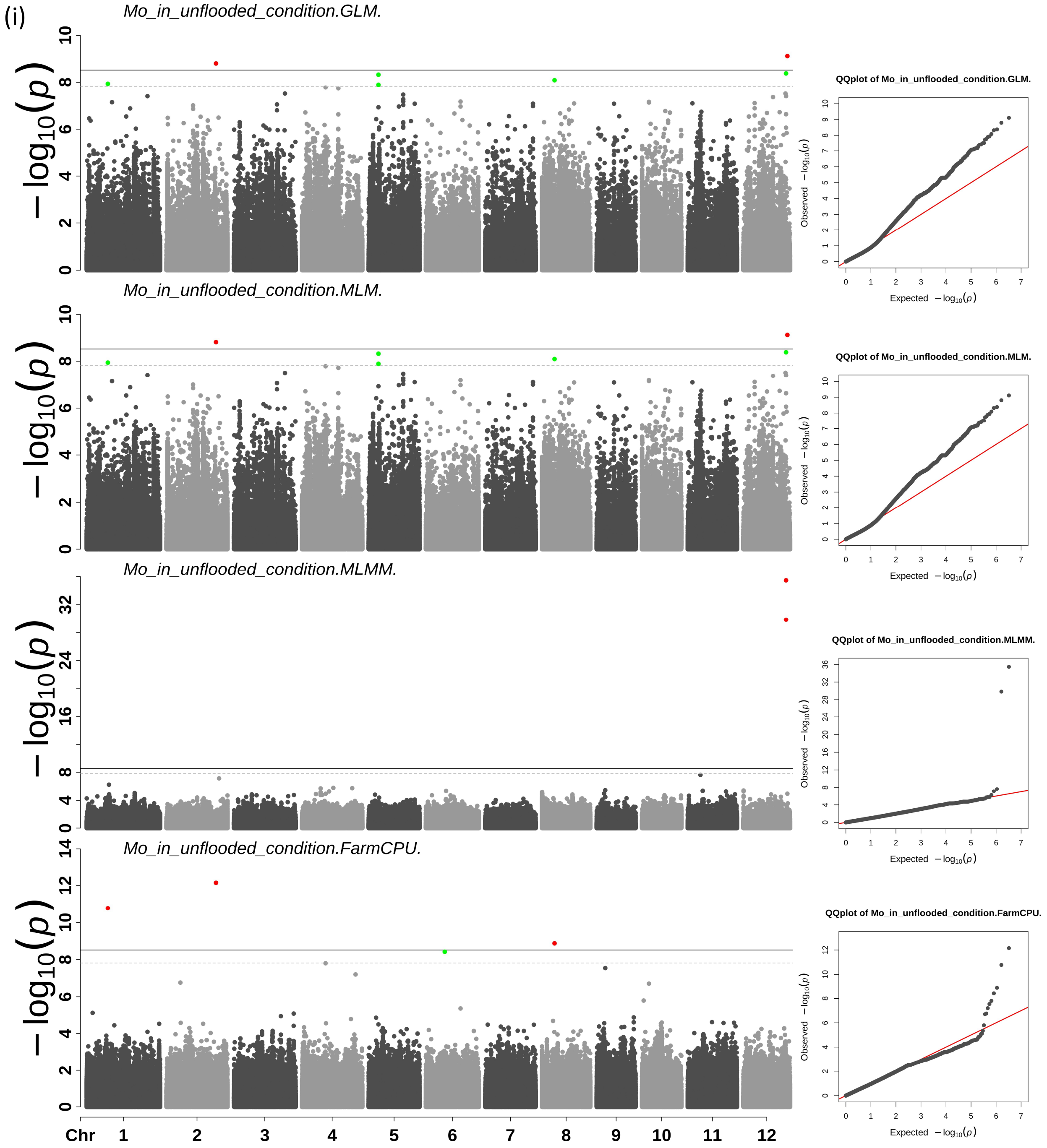


**Supplementary Figure 2 (i)** Genome-wide association analysis for Mo with GLM, MLM, MLMM, and FarmCPU methods (left) in unflooded condition. Quantile-quantile plot of each model (right). The horizontal dot grey line and green dots indicate the Bonferroni-corrected significance thresholds and SNPs at −log10(*P*) = 7.81. The horizontal solid grey line and red dots indicate the Bonferroni-corrected significance thresholds and SNPs at −log10(*P*) = 8.51.

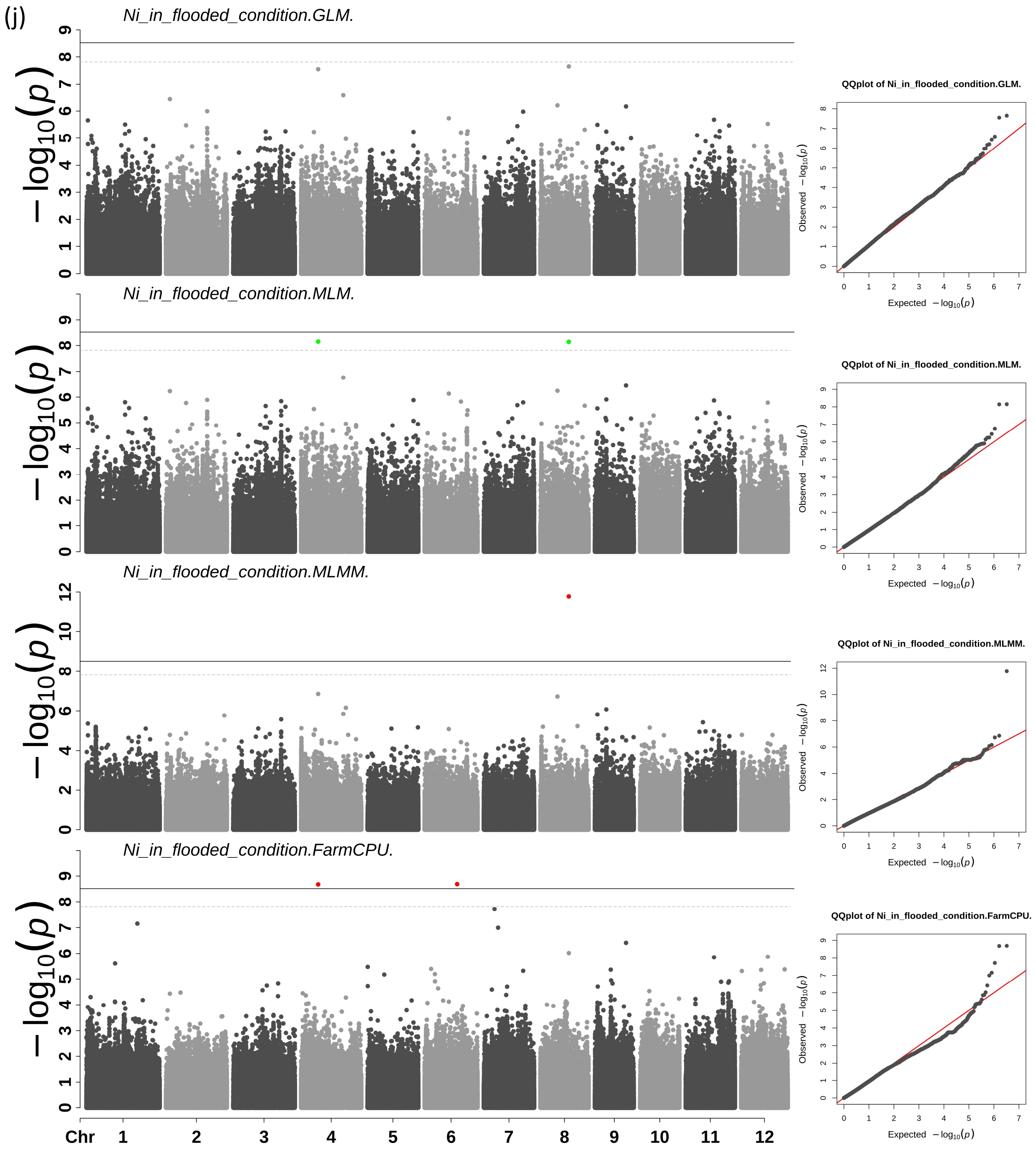


**Supplementary Figure 2 (j)** Genome-wide association analysis for Ni with GLM, MLM, MLMM, and FarmCPU methods (left) in flooded condition. Quantile-quantile plot of each model (right). The horizontal dot grey line and green dots indicate the Bonferroni-corrected significance thresholds and SNPs at −log10(*P*) = 7.81. The horizontal solid grey line and red dots indicate the Bonferroni-corrected significance thresholds and SNPs at −log10(*P*) = 8.51.

(k)

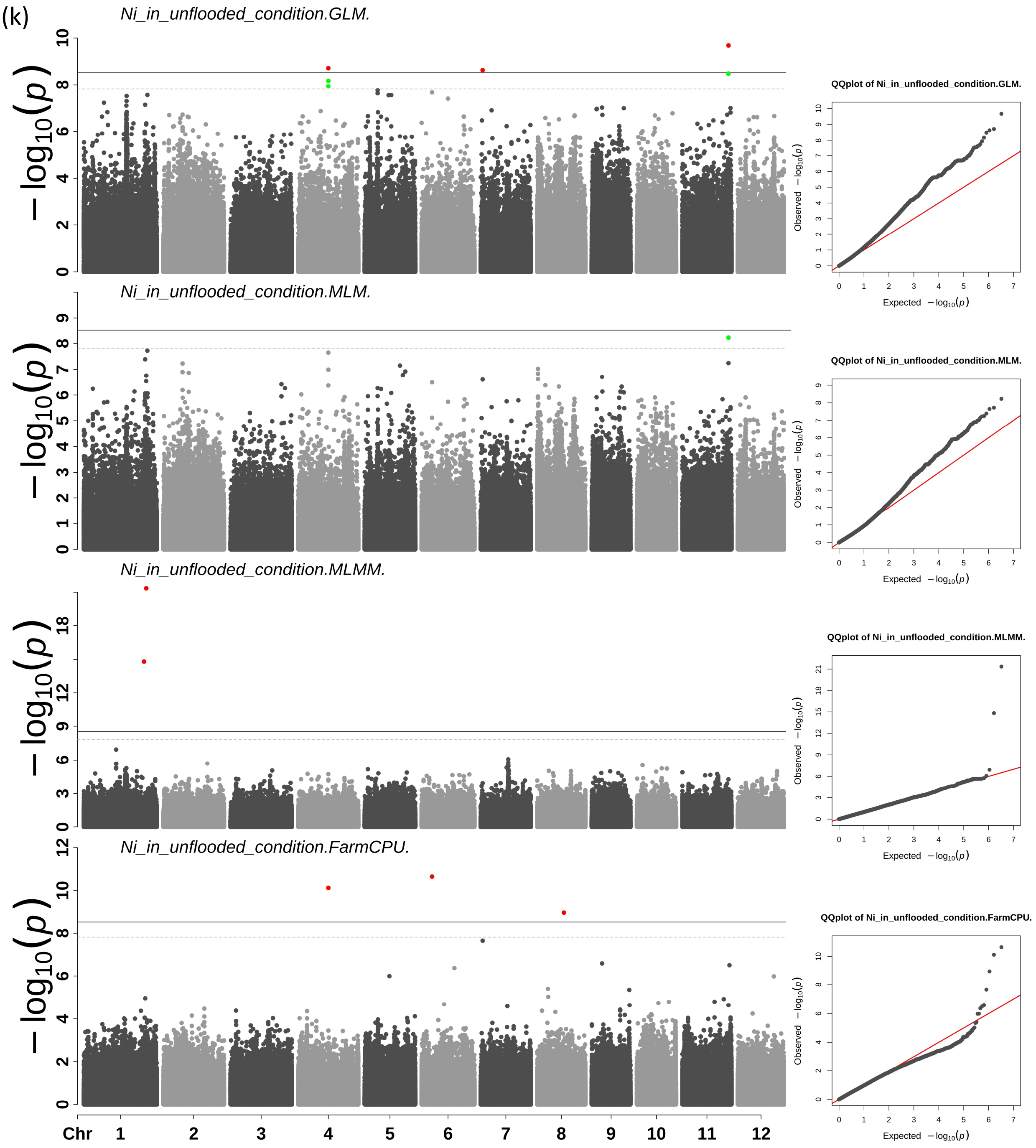


**Supplementary Figure 2 (k)** Genome-wide association analysis for Ni with GLM, MLM, MLMM, and FarmCPU methods (left) in unflooded condition. Quantile-quantile plot of each model (right). The horizontal dot grey line and green dots indicate the Bonferroni-corrected significance thresholds and SNPs at −log10(*P*) = 7.81. The horizontal solid grey line and red dots indicate the Bonferroni-corrected significance thresholds and SNPs at −log10(*P*) = 8.51.

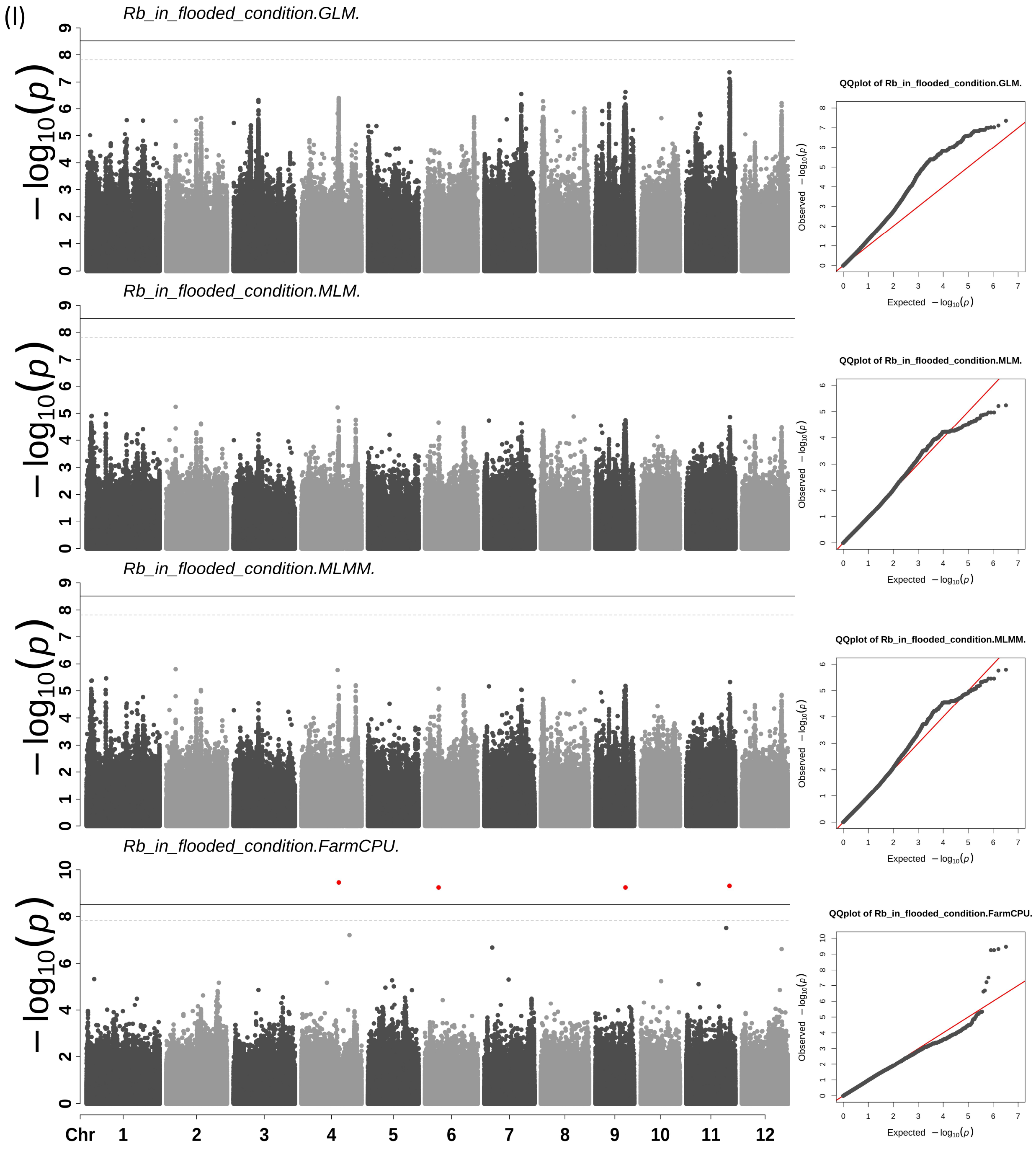


**Supplementary Figure 2 (l)** Genome-wide association analysis for Rb with GLM, MLM, MLMM, and FarmCPU methods (left) in flooded condition. Quantile-quantile plot of each model (right). The horizontal dot grey line and green dots indicate the Bonferroni-corrected significance thresholds and SNPs at −log10(*P*) = 7.81. The horizontal solid grey line and red dots indicate the Bonferroni-corrected significance thresholds and SNPs at −log10(*P*) = 8.51.

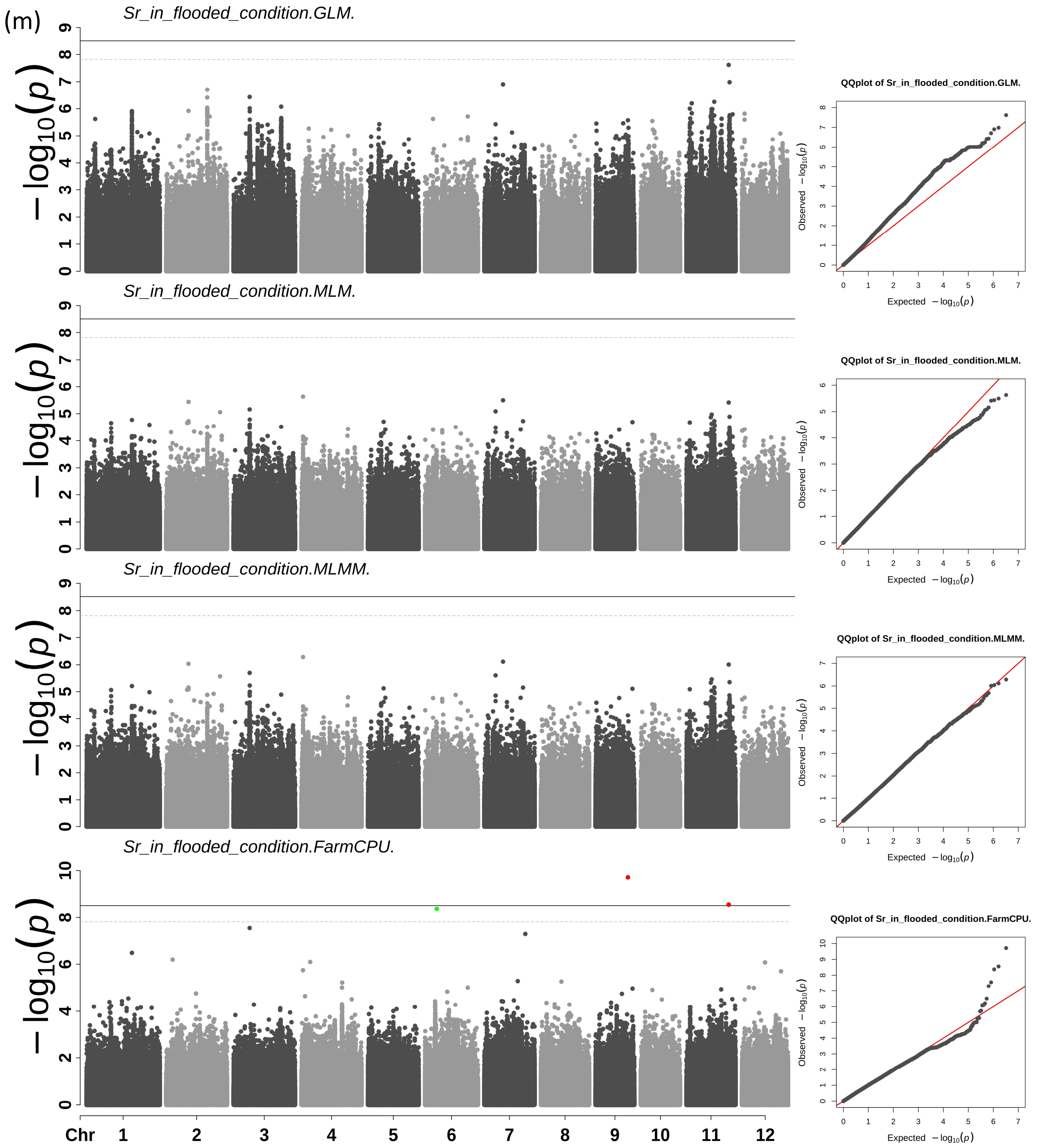


**Supplementary Figure 2 (m)** Genome-wide association analysis for Sr with GLM, MLM, MLMM, and FarmCPU methods (left) in flooded condition. Quantile-quantile plot of each model (right). The horizontal dot grey line and green dots indicate the Bonferroni-corrected significance thresholds and SNPs at −log10(*P*) = 7.81. The horizontal solid grey line and red dots indicate the Bonferroni-corrected significance thresholds and SNPs at −log10(*P*) = 8.51.

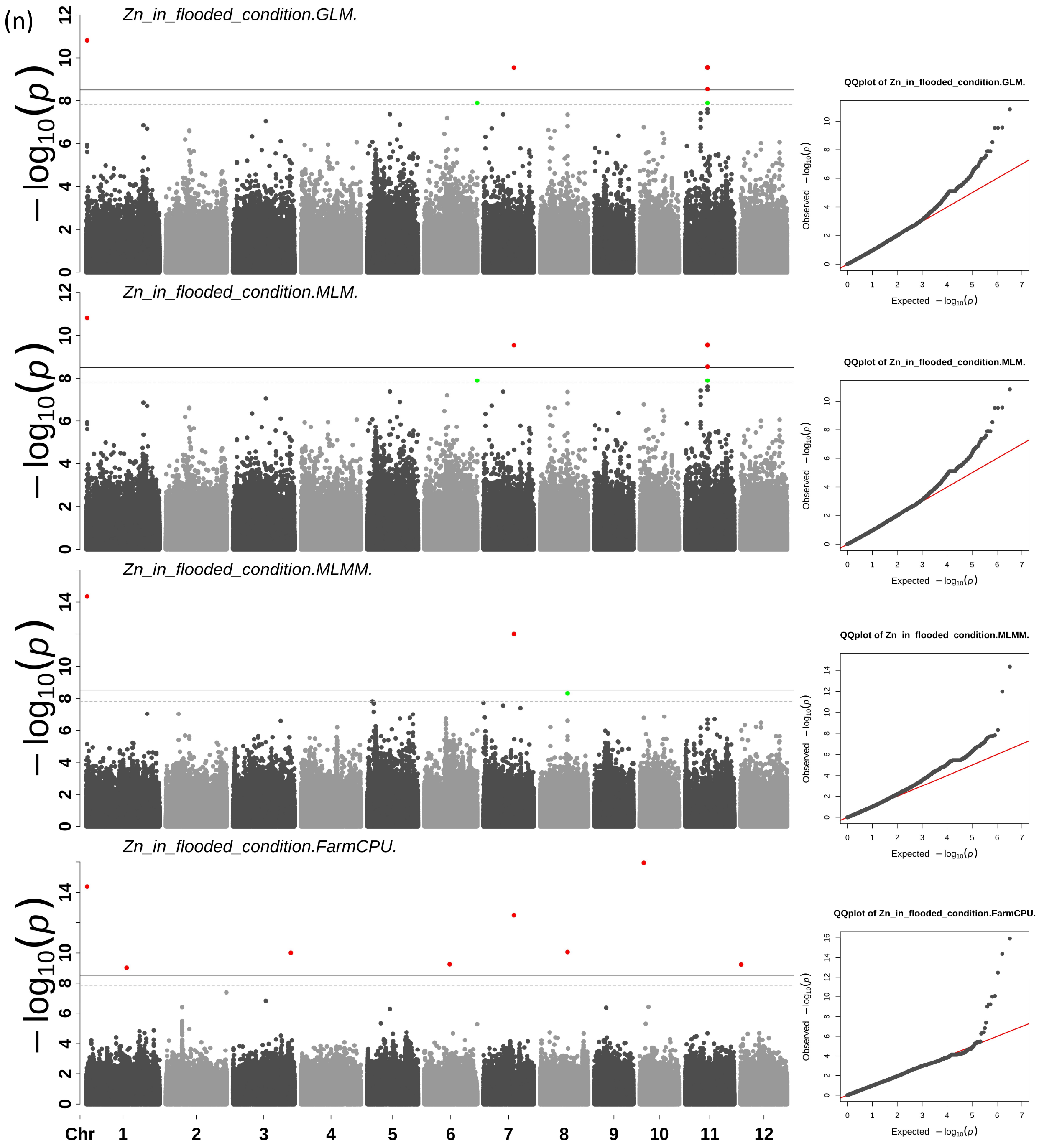


**Supplementary Figure 2 (n)** Genome-wide association analysis for Zn with GLM, MLM, MLMM, and FarmCPU methods (left) in flooded condition. Quantile-quantile plot of each model (right). The horizontal dot grey line and green dots indicate the Bonferroni-corrected significance thresholds and SNPs at −log10(*P*) = 7.81. The horizontal solid grey line and red dots indicate the Bonferroni-corrected significance thresholds and SNPs at −log10(*P*) = 8.51.

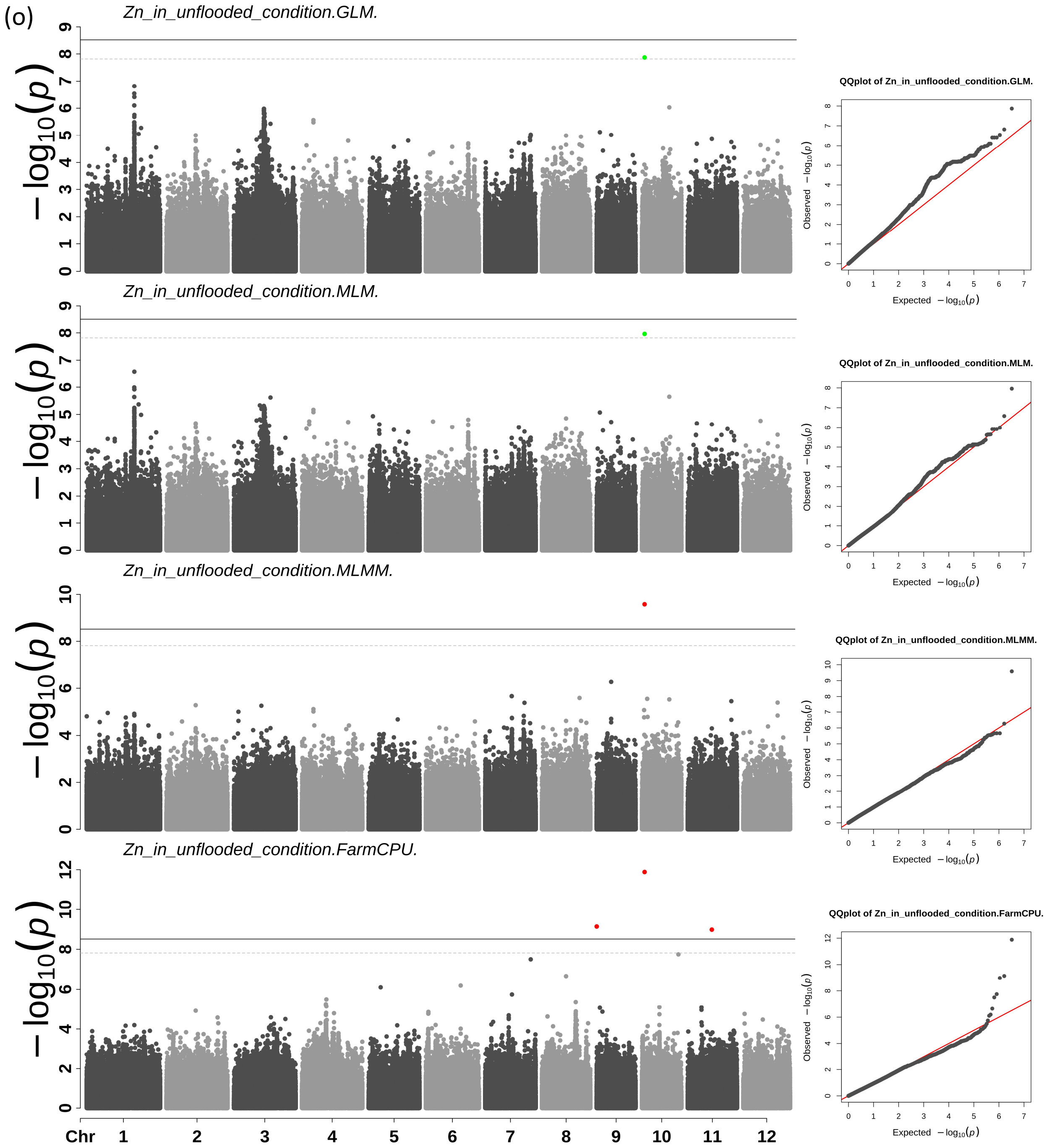


**Supplementary Figure 2 (o)** Genome-wide association analysis for Zn with GLM, MLM, MLMM, and FarmCPU methods (left) in unflooded condition. Quantile-quantile plot of each model (right). The horizontal dot grey line and green dots indicate the Bonferroni-corrected significance thresholds and SNPs at −log10(*P*) = 7.81. The horizontal solid grey line and red dots indicate the Bonferroni-corrected significance thresholds and SNPs at −log10(*P*) = 8.51.
