# Supplementary material for: Genome-wide association studies of ionomic and agronomic traits in USDA mini core collection of rice and comparative analyses of different mapping methods
Source: BMC Plant Biol. 2020 Sep 24;20:441. doi: 10.1186/s12870-020-02603-0 (PMC7513512; doi:10.1186/s12870-020-02603-0)
Supplement: Supplementary file 3 — Additional file 3: Supplementary Figure 3. (a) Genome-wide association analysis for AMYLOSE with GLM, MLM, MLMM, and FarmCPU methods (left). Quantile-quantile plot of each model (right). Red arrows indicate published genes. The horizontal dot grey line and green dots indicate the Bonferroni-corrected significance thresholds and SNPs at −log10(P) = 7.81. The horizontal solid grey line and red dots indicate the Bonferroni-corrected significance thresholds and SNPs at −log10(P) = 8.51. (b) Genome-wide association analysis for AWNTYPE with GLM, MLM, MLMM, and FarmCPU methods (left). Quantile-quantile plot of each model (right). The horizontal dot grey line and green dots indicate the Bonferroni-corrected significance thresholds and SNPs at −log10(P) = 7.81. The horizontal solid grey line and red dots indicate the Bonferroni-corrected significance thresholds and SNPs at −log10(P) = 8.51. (c) Genome-wide association analysis for DAYSFLOWER with GLM, MLM, MLMM, and FarmCPU methods (left). Quantile-quantile plot of each model (right). Black arrow indicates candidate gene. The horizontal dot grey line and green dots indicate the Bonferroni-corrected significance thresholds and SNPs at −log10(P) = 7.81. The horizontal solid grey line and red dots indicate the Bonferroni-corrected significance thresholds and SNPs at −log10(P) = 8.51. (d) Genome-wide association analysis for HULLCOLOR with GLM, MLM, MLMM, and FarmCPU methods (left). Quantile-quantile plot of each model (right). The horizontal dot grey line and green dots indicate the Bonferroni-corrected significance thresholds and SNPs at −log10(P) = 7.81. The horizontal solid grey line and red dots indicate the Bonferroni-corrected significance thresholds and SNPs at −log10(P) = 8.51. (e) Genome-wide association analysis for HULLCOVER with GLM, MLM, MLMM, and FarmCPU methods (left). Quantile-quantile plot of each model (right). The horizontal dot grey line and green dots indicate the Bonferroni-corrected significance threshol [file 12870_2020_2603_MOESM3_ESM.pdf]

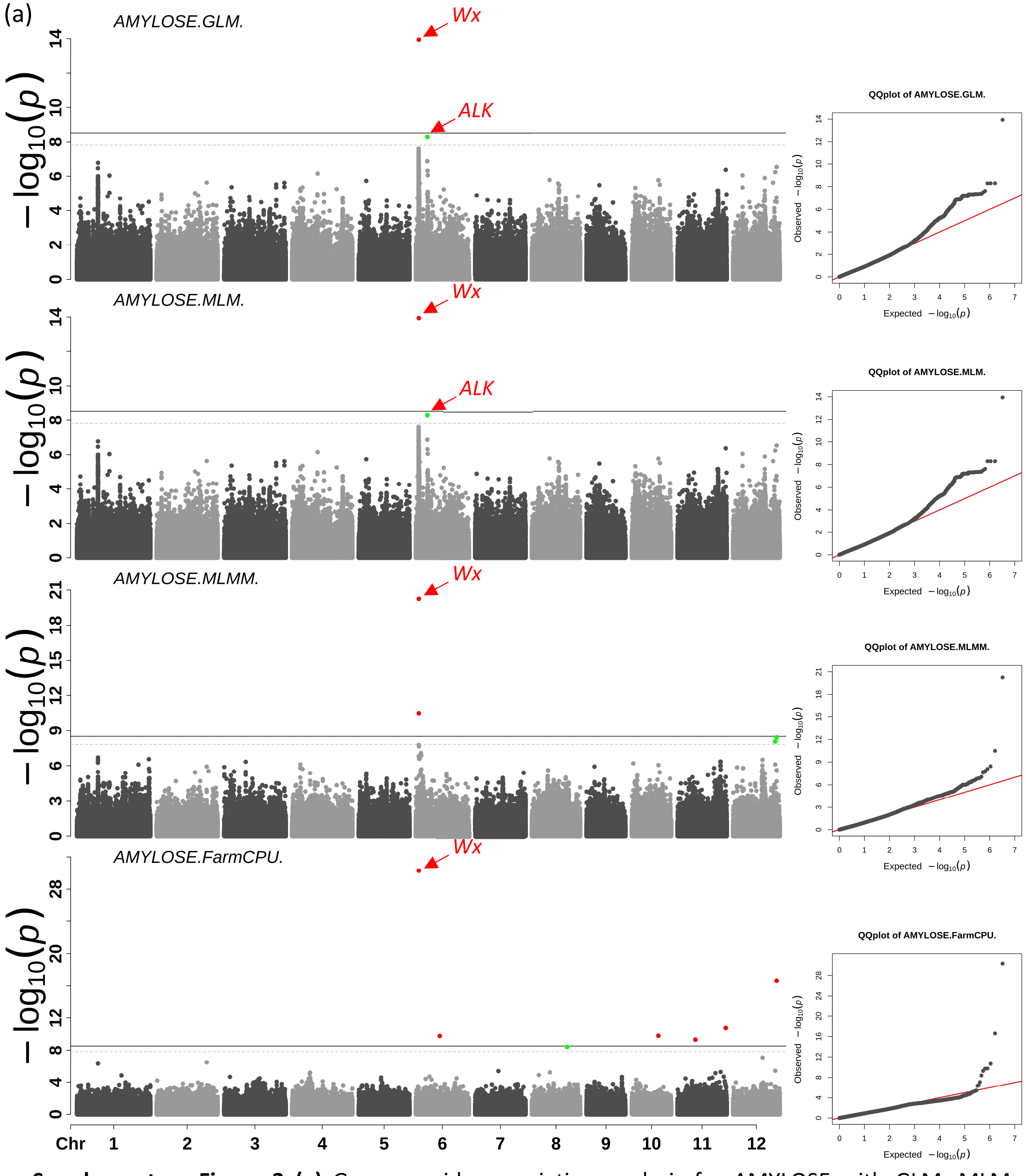

Supplementary Figure 3 (a) Genome-wide association analysis for AMYLOSE with GLM, MLM, MLMM, and FarmCPU methods (left). Quantile-quantile plot of each model (right). Red arrows indicate published genes. The horizontal dot grey line and green dots indicate the Bonferroni-corrected significance thresholds and SNPs at −log10(*P*) = 7.81. The horizontal solid grey line and red dots indicate the Bonferroni-corrected significance thresholds and SNPs at −log10(*P*) = 8.51.

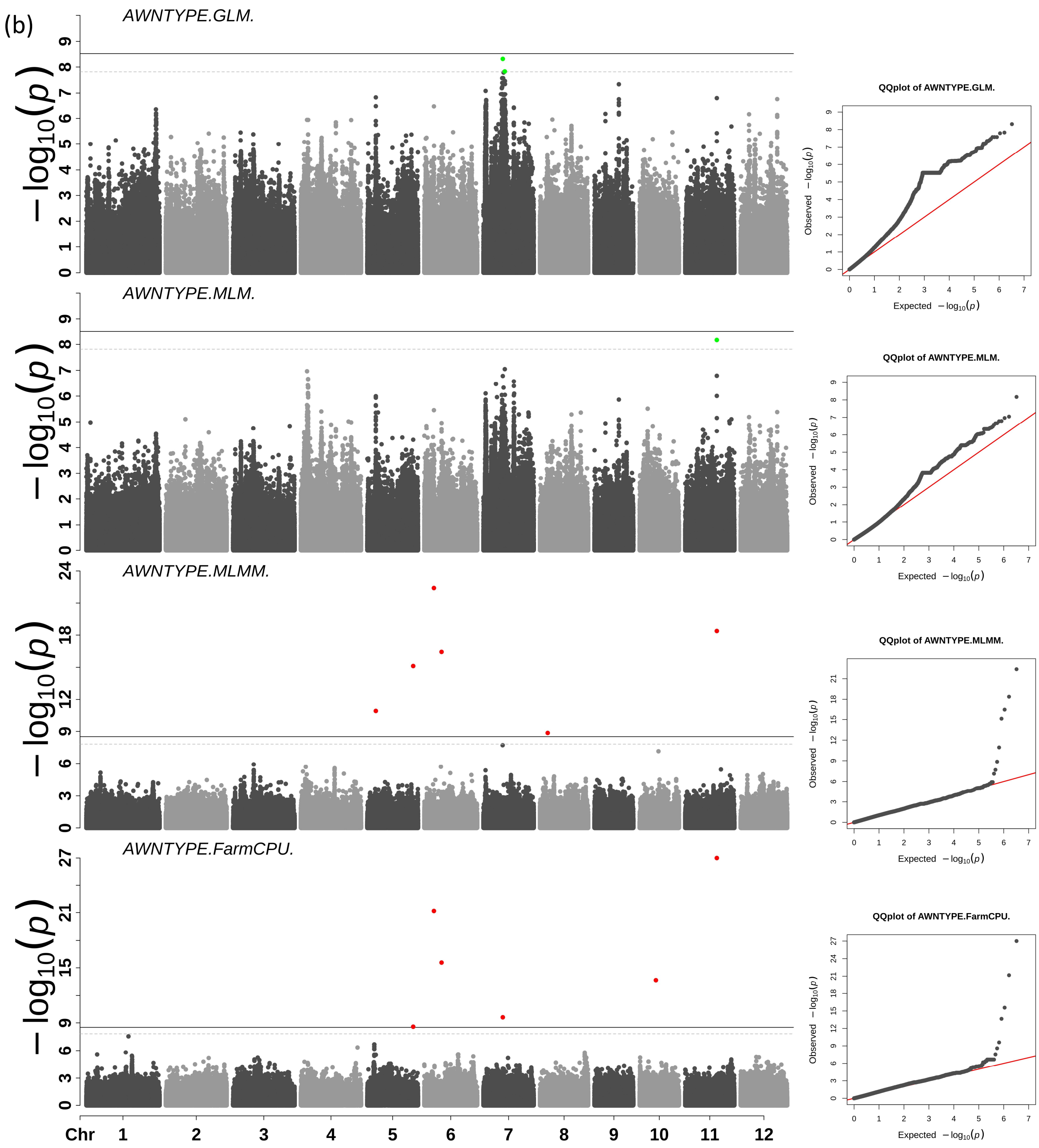

Supplementary Figure 3 (b) Genome-wide association analysis for AWNTYPE with GLM, MLM, MLMM, and FarmCPU methods (left). Quantile-quantile plot of each model (right). The horizontal dot grey line and green dots indicate the Bonferroni-corrected significance thresholds and SNPs at −log10(*P*) = 7.81. The horizontal solid grey line and red dots indicate the Bonferroni-corrected significance thresholds and SNPs at −log10(*P*) = 8.51.

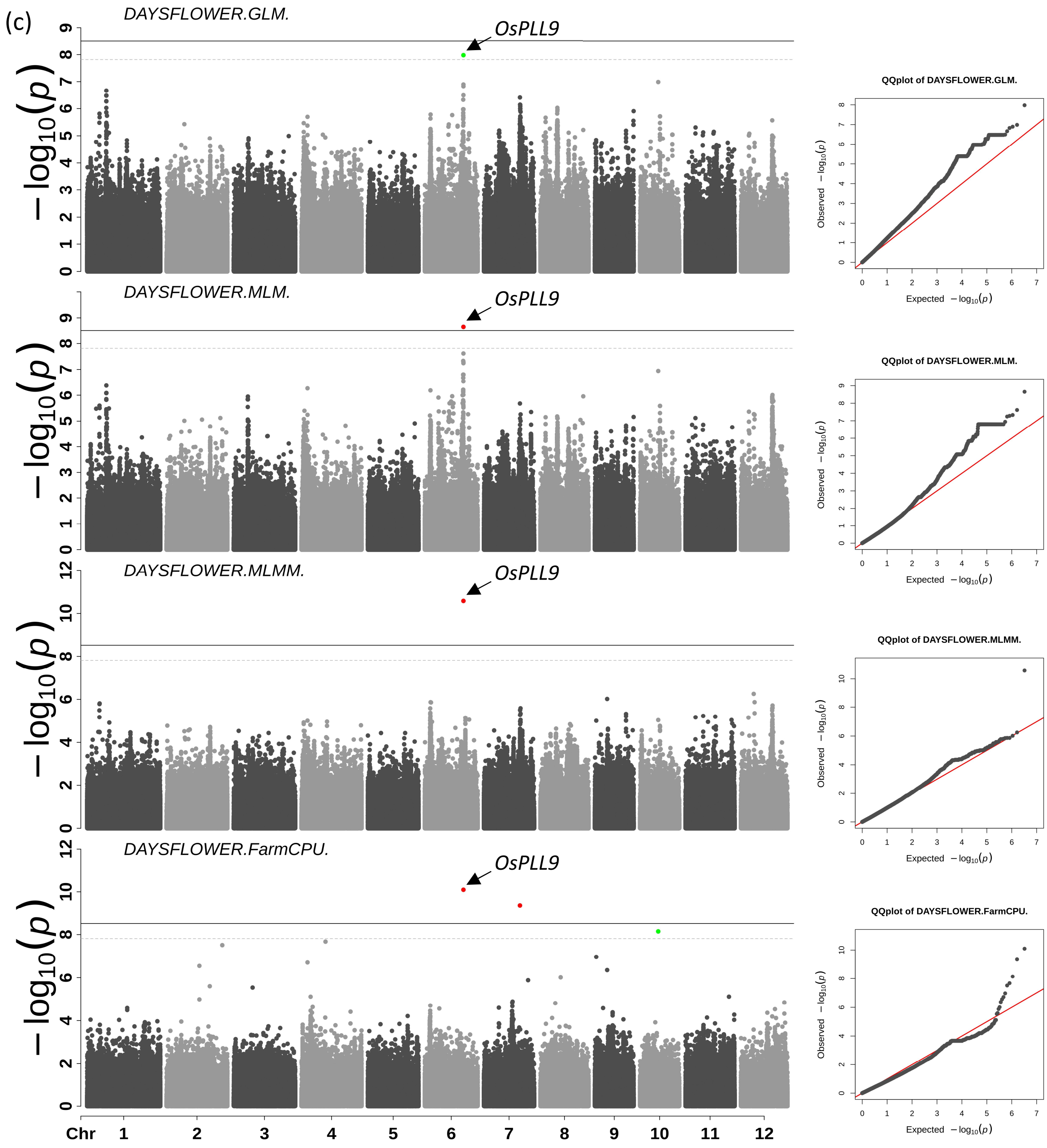

Supplementary Figure 3 (c) Genome-wide association analysis for DAYSFLOWER with GLM, MLM, MLMM, and FarmCPU methods (left). Quantile-quantile plot of each model (right). Black arrow indicates candidate gene. The horizontal dot grey line and green dots indicate the Bonferroni-corrected significance thresholds and SNPs at −log10(*P*) = 7.81. The horizontal solid grey line and red dots indicate the Bonferroni-corrected significance thresholds and SNPs at −log10(*P*) = 8.51.

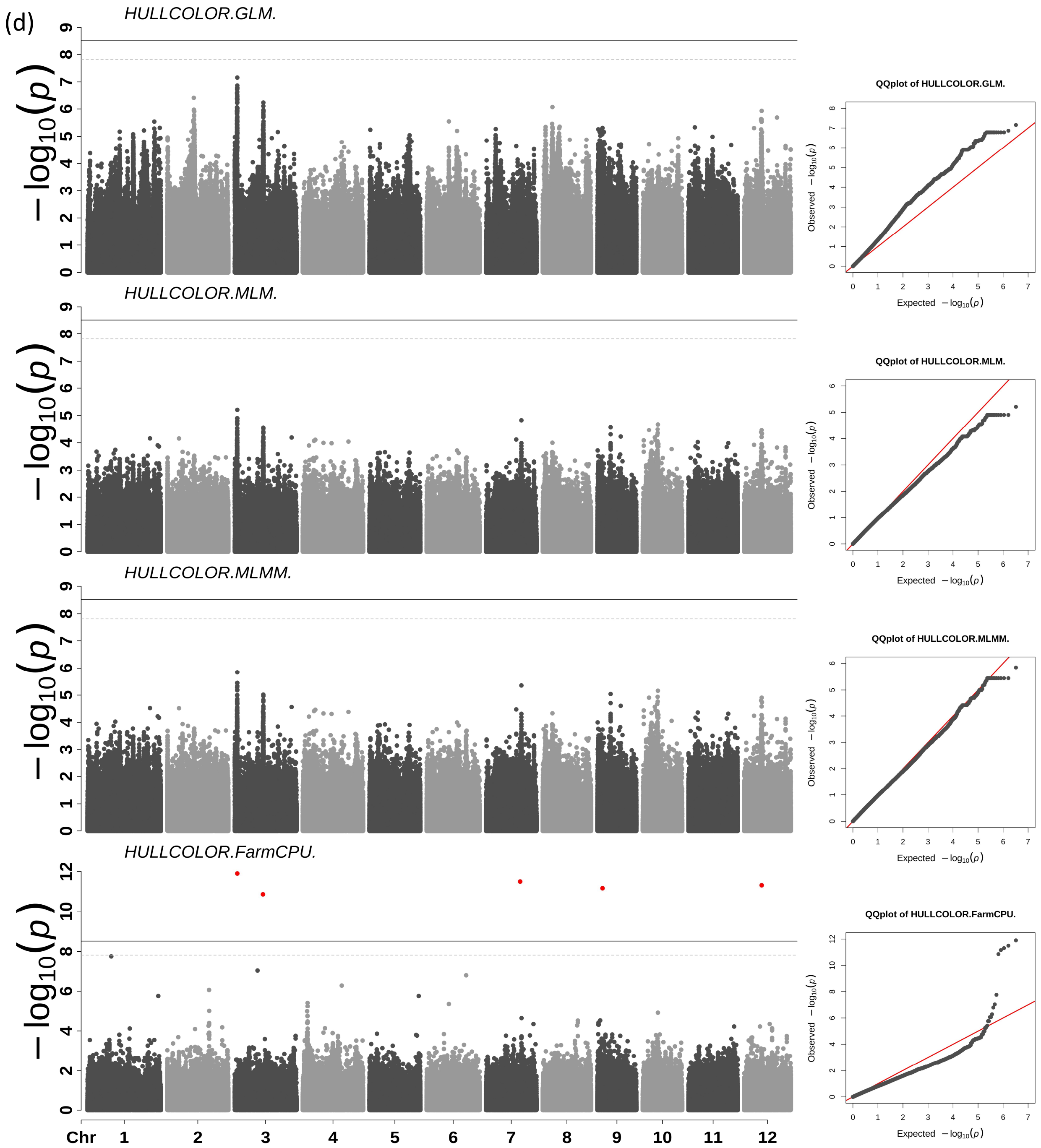

Supplementary Figure 3 (d) Genome-wide association analysis for HULLCOLOR with GLM, MLM, MLMM, and FarmCPU methods (left). Quantile-quantile plot of each model (right). The horizontal dot grey line and green dots indicate the Bonferroni-corrected significance thresholds and SNPs at −log10(*P*) = 7.81. The horizontal solid grey line and red dots indicate the Bonferroni-corrected significance thresholds and SNPs at −log10(*P*) = 8.51.

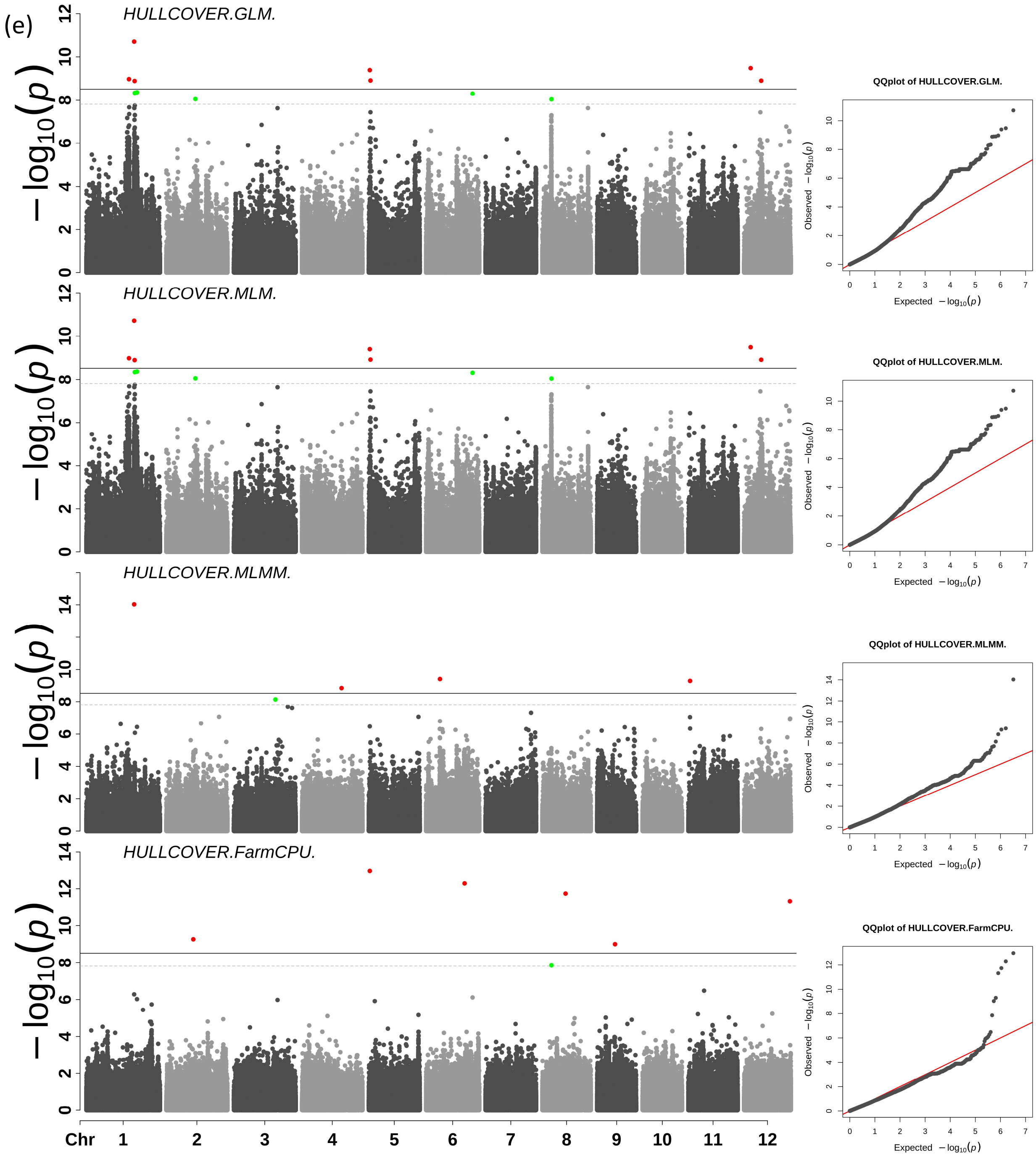

Supplementary Figure 3 (e) Genome-wide association analysis for HULLCOVER with GLM, MLM, MLMM, and FarmCPU methods (left). Quantile-quantile plot of each model (right). The horizontal dot grey line and green dots indicate the Bonferroni-corrected significance thresholds and SNPs at −log10(*P*) = 7.81. The horizontal solid grey line and red dots indicate the Bonferroni-corrected significance thresholds and SNPs at −log10(*P*) = 8.51.

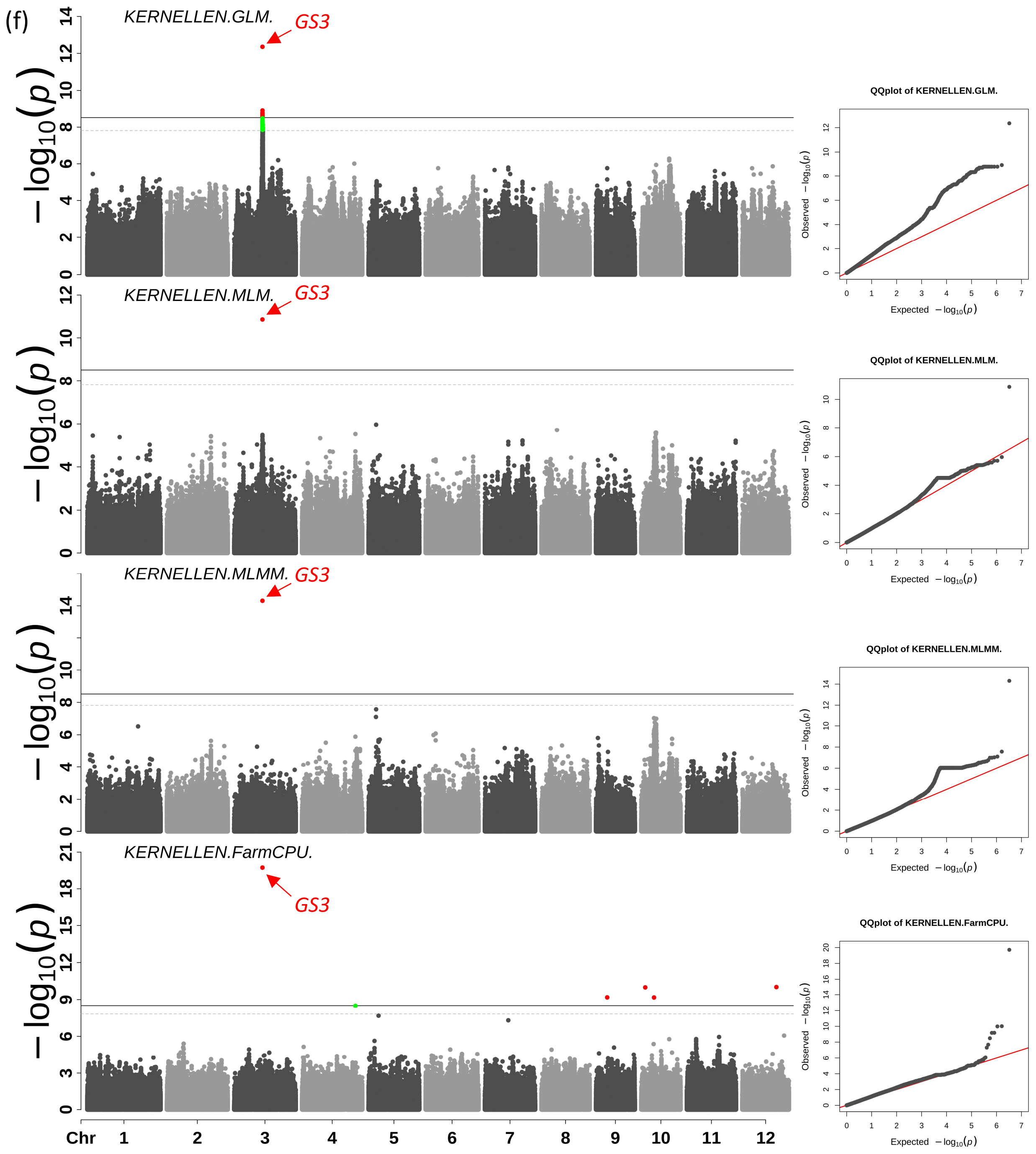

Supplementary Figure 3 (f) Genome-wide association analysis for KERNELLEN with GLM, MLM, MLMM, and FarmCPU methods (left). Quantile-quantile plot of each model (right). Red arrow indicates published gene. The horizontal dot grey line and green dots indicate the Bonferroni-corrected significance thresholds and SNPs at −log10(*P*) = 7.81. The horizontal solid grey line and red dots indicate the Bonferroni-corrected significance thresholds and SNPs at −log10(*P*) = 8.51.

(g)

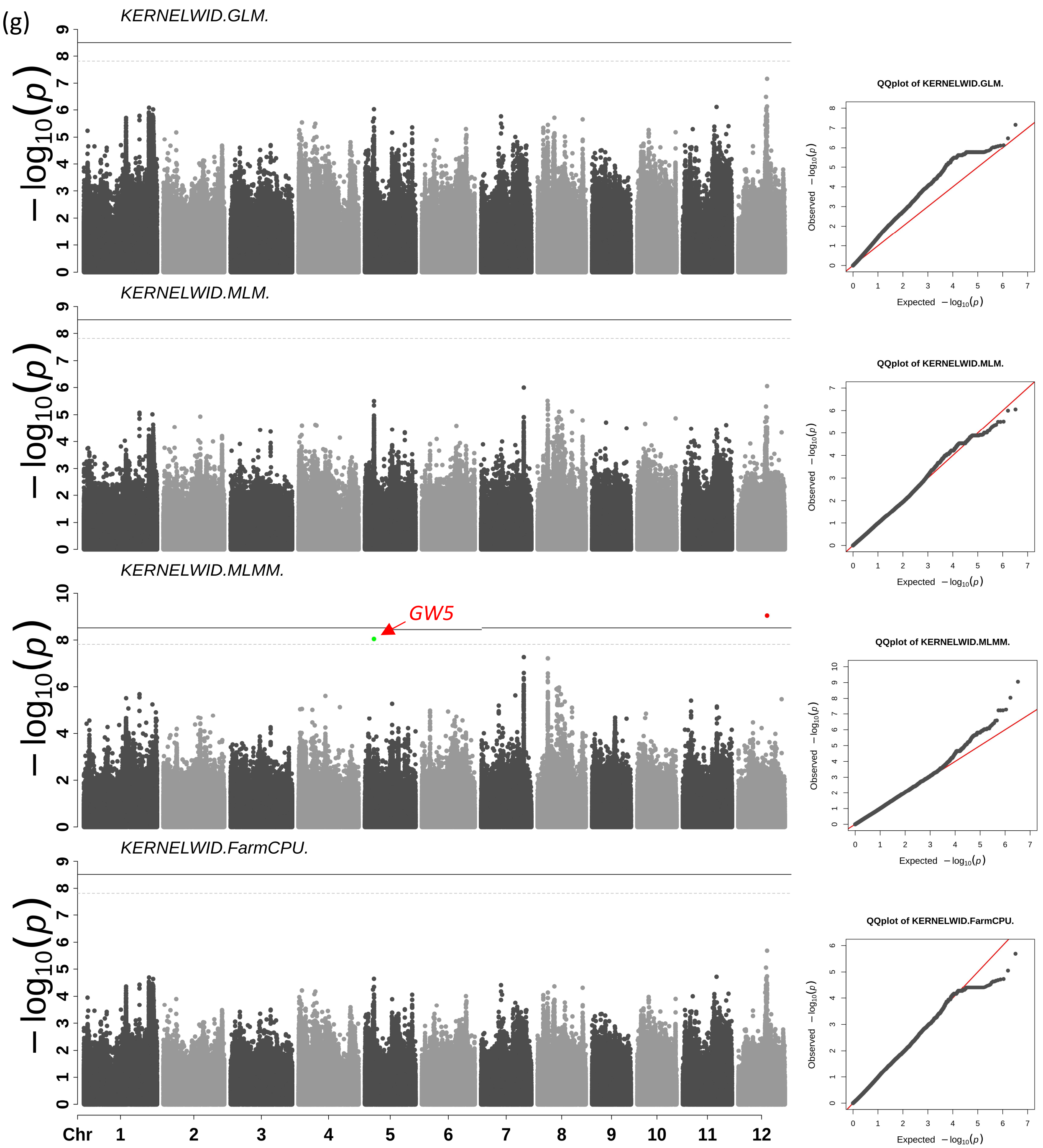

Supplementary Figure 3 (g) Genome-wide association analysis for KERNELWID with GLM, MLM, MLMM, and FarmCPU methods (left). Quantile-quantile plot of each model (right). Red arrow indicates published gene. The horizontal dot grey line and green dots indicate the Bonferroni-corrected significance thresholds and SNPs at −log10(*P*) = 7.81. The horizontal solid grey line and red dots indicate the Bonferroni-corrected significance thresholds and SNPs at −log10(*P*) = 8.51.

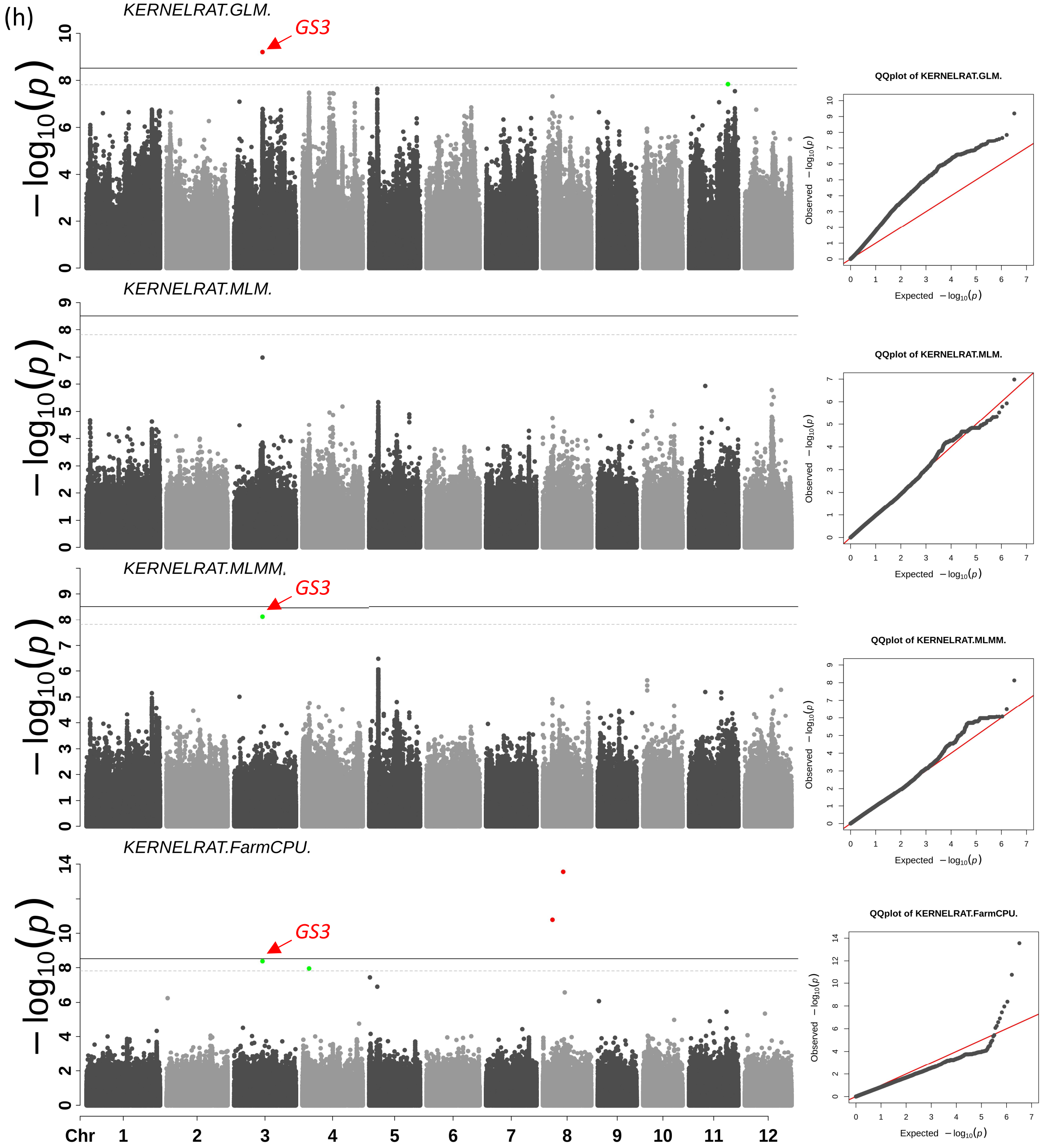

Supplementary Figure 3 (h) Genome-wide association analysis for KERNELRAT with GLM, MLM, MLMM, and FarmCPU methods (left). Quantile-quantile plot of each model (right). Red arrow indicates published gene. The horizontal dot grey line and green dots indicate the Bonferroni-corrected significance thresholds and SNPs at −log10(*P*) = 7.81. The horizontal solid grey line and red dots indicate the Bonferroni-corrected significance thresholds and SNPs at −log10(*P*) = 8.51.

(i)

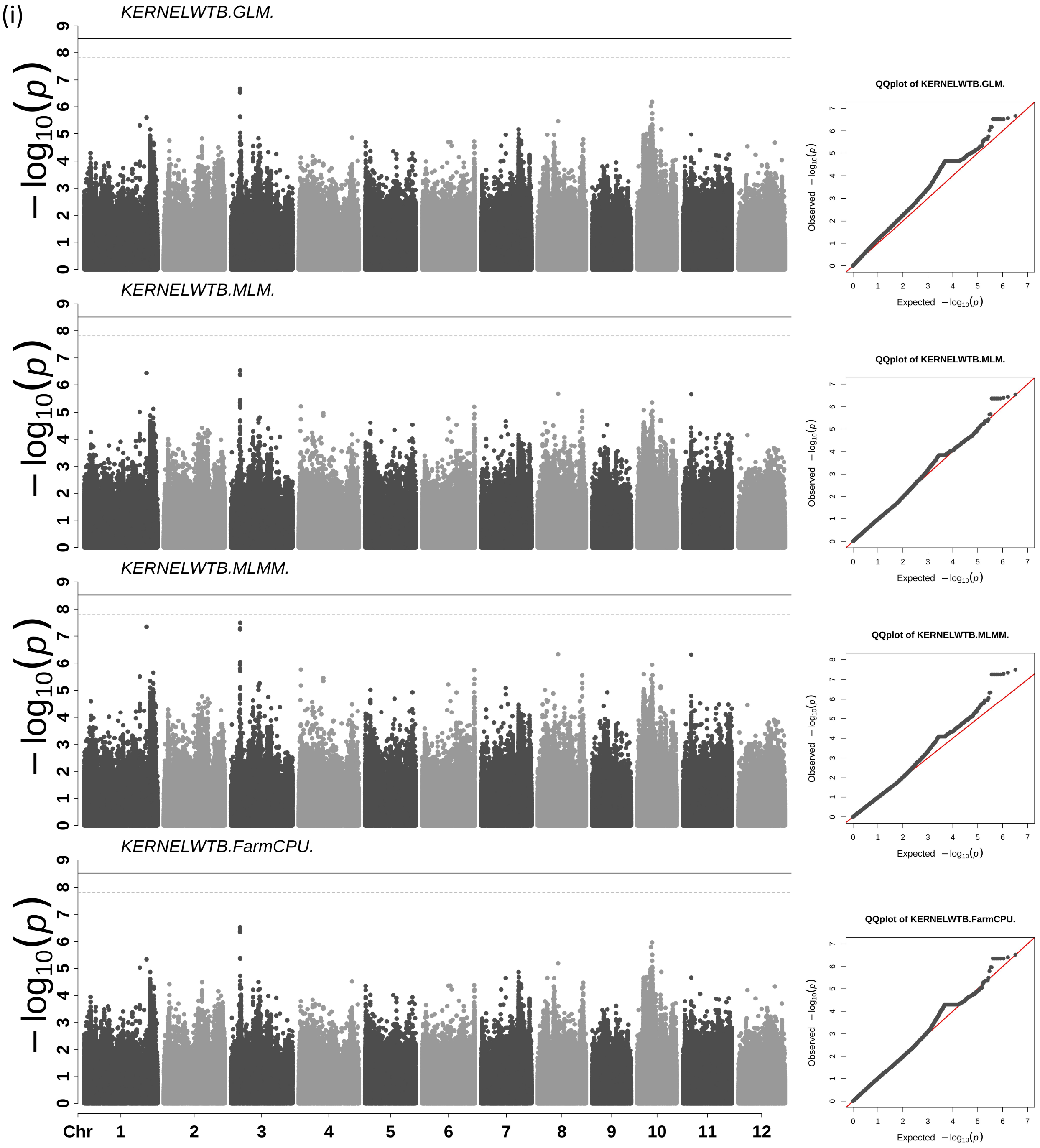

Supplementary Figure 3 (i) Genome-wide association analysis for KERNELWTB with GLM, MLM, MLMM, and FarmCPU methods (left). Quantile-quantile plot of each model (right). The horizontal dot grey line and green dots indicate the Bonferroni-corrected significance thresholds and SNPs at −log10(*P*) = 7.81. The horizontal solid grey line and red dots indicate the Bonferroni-corrected significance thresholds and SNPs at −log10(*P*) = 8.51.

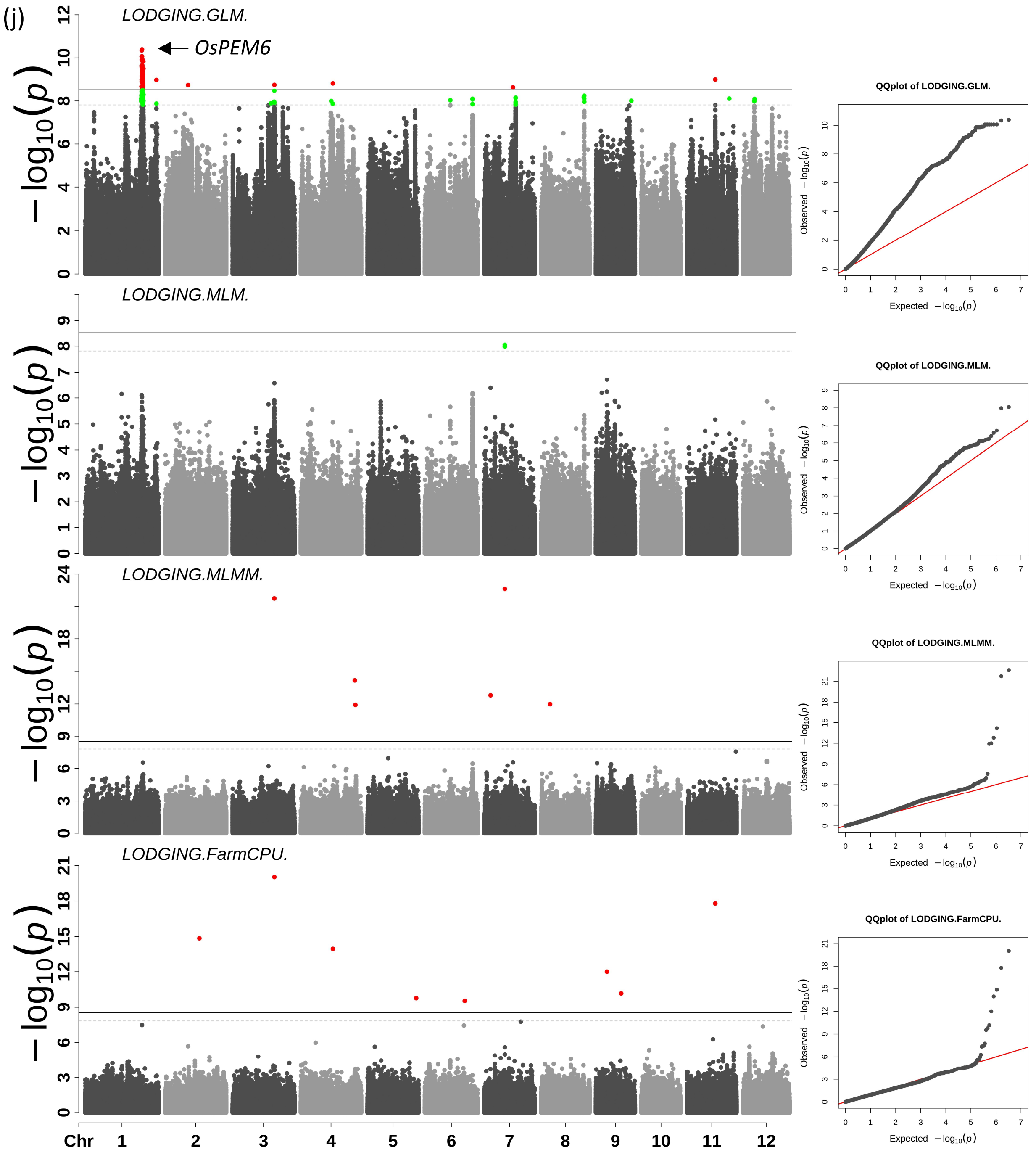

Supplementary Figure 3 (j) Genome-wide association analysis for LODGING with GLM, MLM, MLMM, and FarmCPU methods (left). Quantile-quantile plot of each model (right). Black arrow indicates candidate gene. The horizontal dot grey line and green dots indicate the Bonferroni-corrected significance thresholds and SNPs at −log10(*P*) = 7.81. The horizontal solid grey line and red dots indicate the Bonferroni-corrected significance thresholds and SNPs at −log10(*P*) = 8.51.

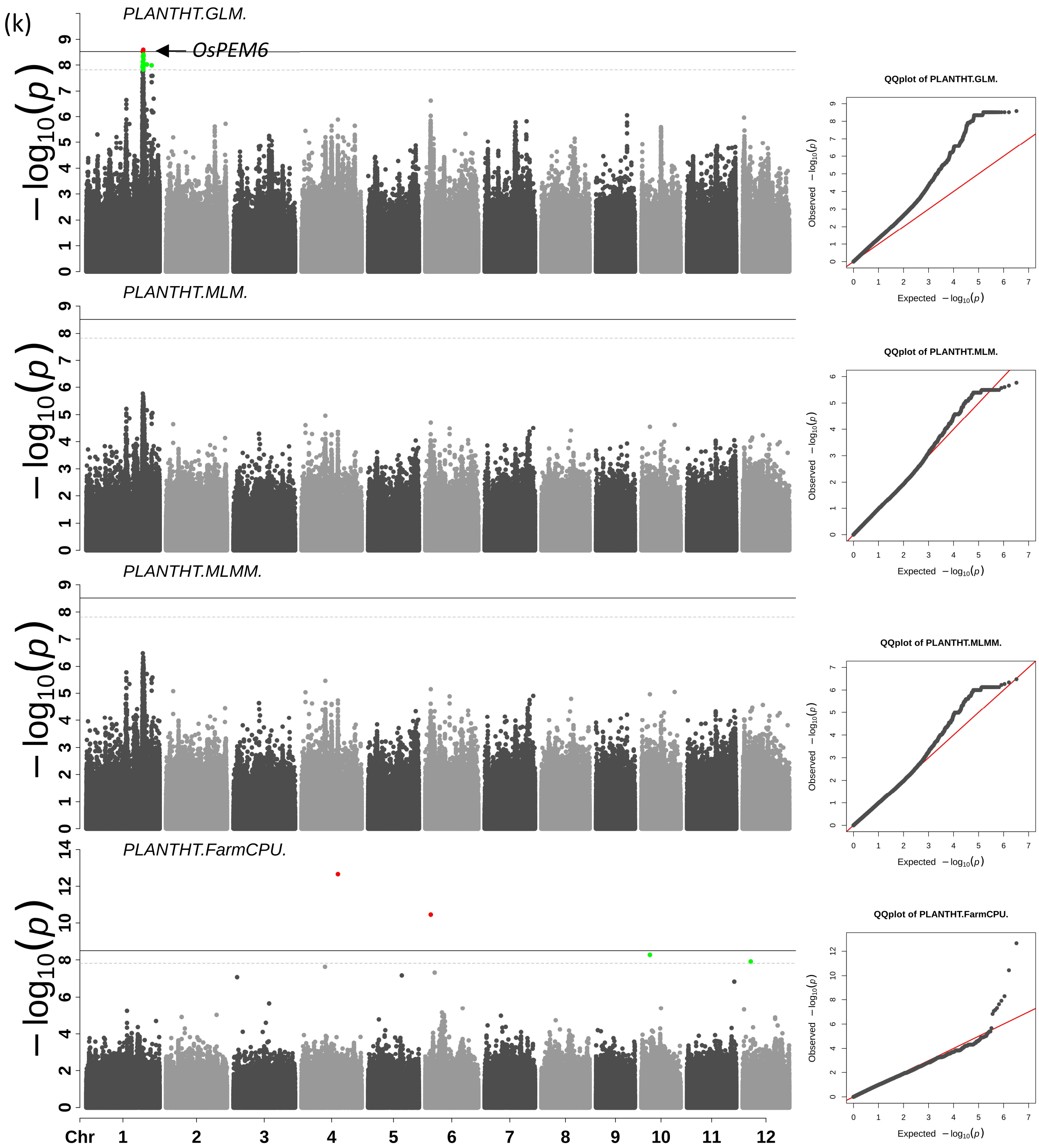

Supplementary Figure 3 (k) Genome-wide association analysis for PLANTHT with GLM, MLM, MLMM, and FarmCPU methods (left). Quantile-quantile plot of each model (right). Black arrow indicates candidate gene. The horizontal dot grey line and green dots indicate the Bonferroni-corrected significance thresholds and SNPs at −log10(*P*) = 7.81. The horizontal solid grey line and red dots indicate the Bonferroni-corrected significance thresholds and SNPs at −log10(*P*) = 8.51.
