# Supplementary figures and images for: Genome-wide association studies of ionomic and agronomic traits in USDA mini core collection of rice and comparative analyses of different mapping methods
Source: BMC Plant Biol. 2020 Sep 24;20:441. doi: 10.1186/s12870-020-02603-0 (PMC7513512; doi:10.1186/s12870-020-02603-0)

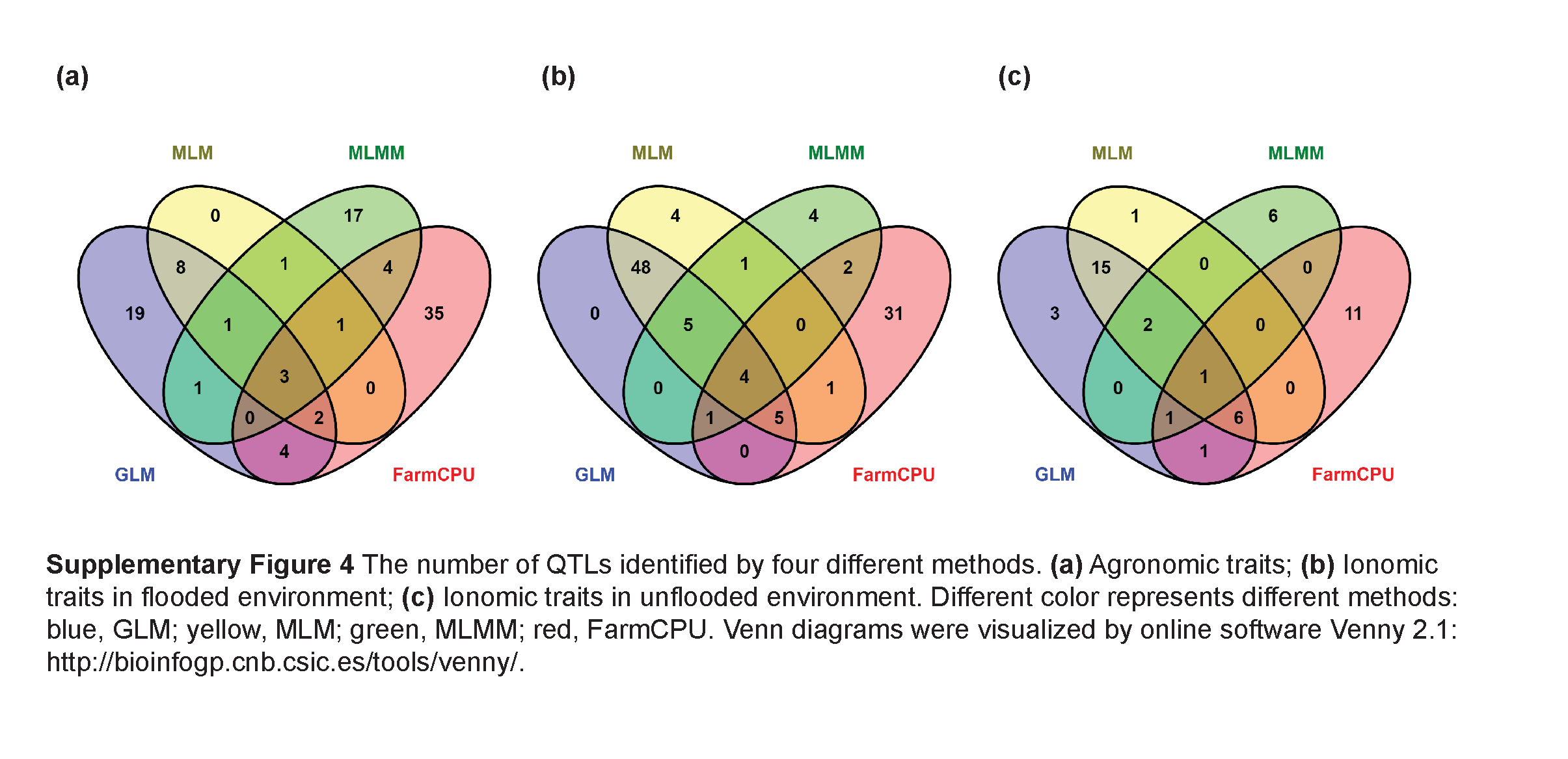

Supplement: Supplementary file 4 — Additional file 4: Supplementary Figure 4. The number of QTLs identified by four different methods. (a) Agronomic traits; (b) Ionomic traits in flooded environment; (c) Ionomic traits in unflooded environment. Different color represents different methods; blue, GLM; yellow, MLM; green, MLMM; red, FarmCPU. Venn diagrams were visualized by online software Venny 2.1: http://bioinfogp.cnb.csic.es/tools/venny/. [file 12870_2020_2603_MOESM4_ESM.tif]

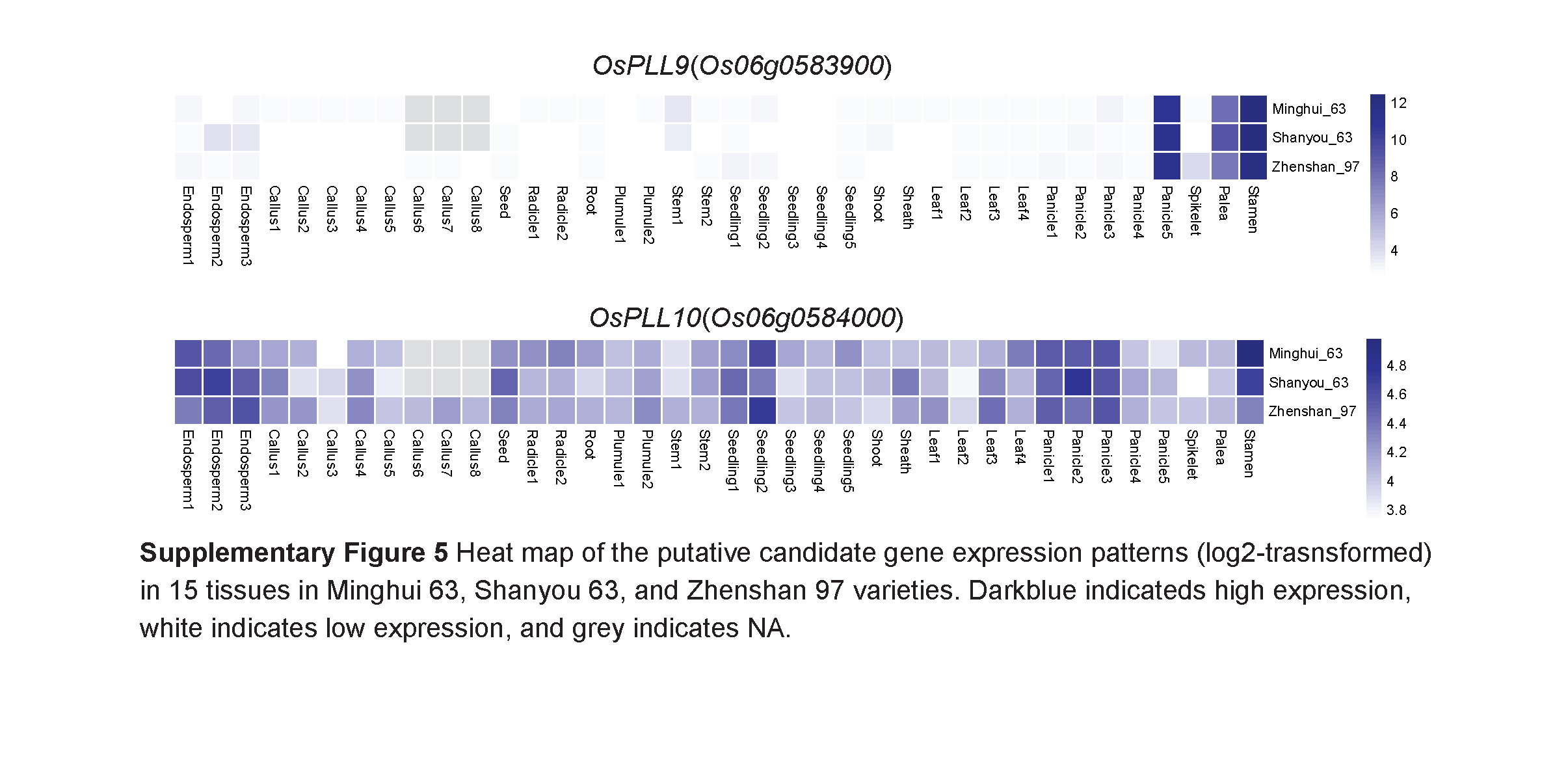

Supplement: Supplementary file 5 — Additional file 5: Supplementary Figure 5. Heat map of the putative candidate gene expression patterns (log2-transformed) in 15 tissues in Minghui 63, Shanyou 63, and Zhenshan 97 varieties. Darkblue indicates high expression, white indicates low expression, and grey indicates NA. [file 12870_2020_2603_MOESM5_ESM.tif]

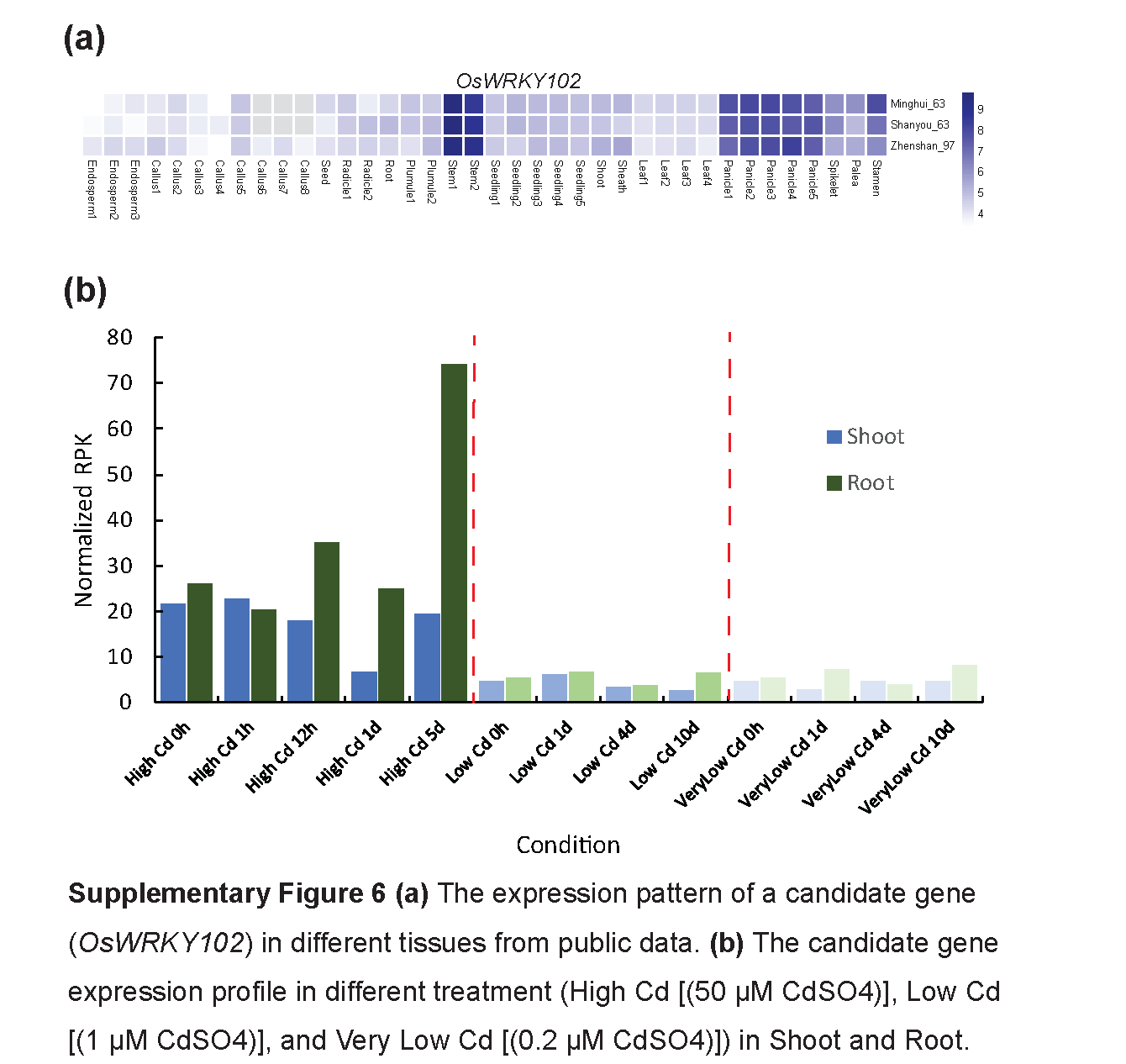

Supplement: Supplementary file 6 — Additional file 6: Supplementary Figure 6. (a) The expression pattern of a candidate gene ( OsWRK102) in different tissues from public data. (b) The candidate gene expression profile in different treatment (High Cd [(50 μM CdSO4)]), Low Cd [(1 μM CdSO4)], and Very Low Cd [(0.2 μM CdSO4)] in Shoot and Root. [file 12870_2020_2603_MOESM6_ESM.tif]

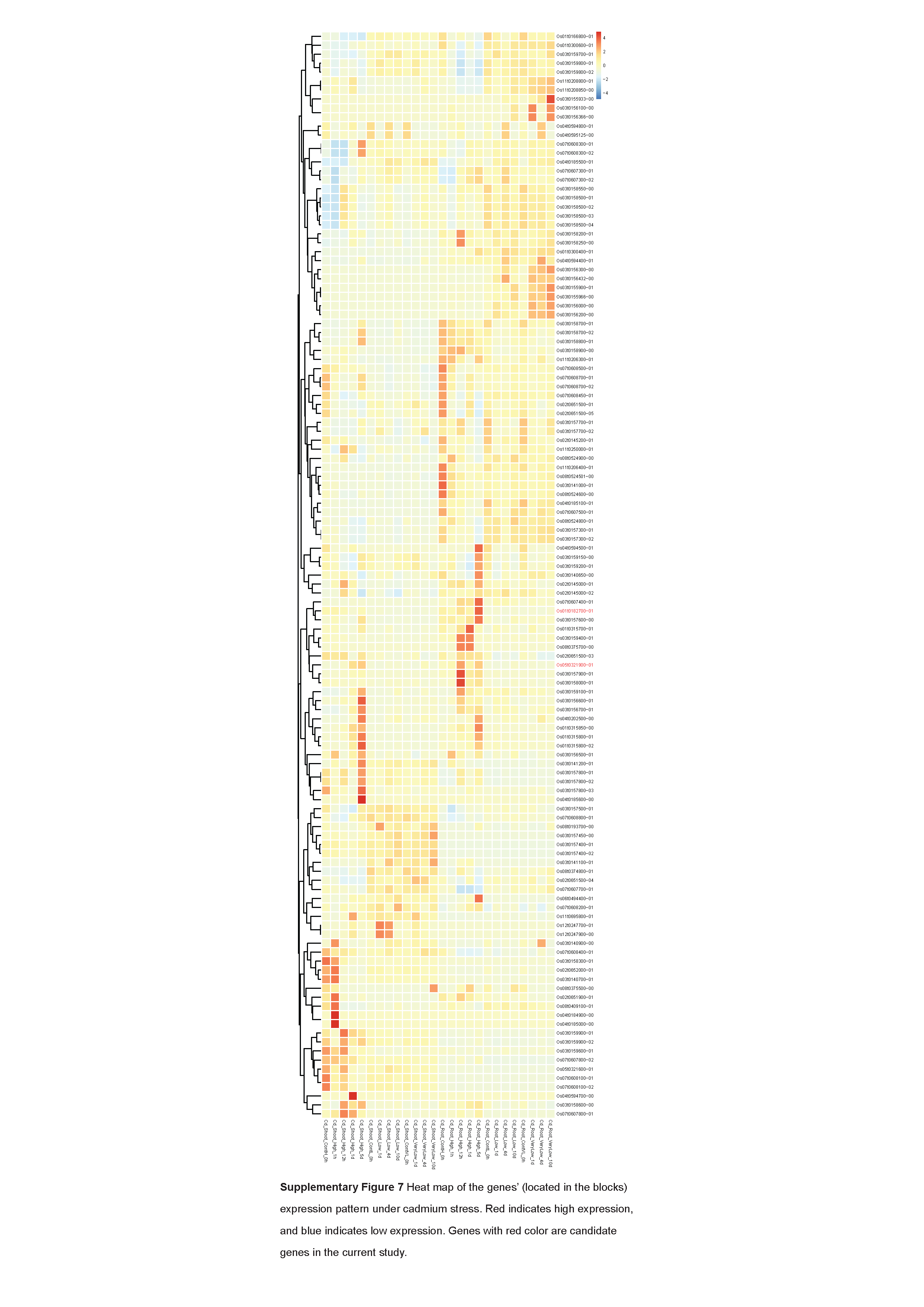

Supplement: Supplementary file 7 — Additional file 7: Supplementary Figure 7. Heat map of the genes’ (located in the blocks) expression patterns under cadmium stress. Red indicates high expression, and blue indicates low expression. Genes with red color are candidate genes in the current study. [file 12870_2020_2603_MOESM7_ESM.tif]
